# Pomegranate extract-loaded sphingosomes for the treatment of cancer: Phytochemical investigations, formulation, and antitumor activity evaluation

**DOI:** 10.1371/journal.pone.0293115

**Published:** 2024-02-12

**Authors:** Huda Jamal AlMadalli, Bazigha K. Abdul Rasool, Naglaa Gamil Shehab, Francesca Della Sala, Assunta Borzacchiello

**Affiliations:** 1 Pharmaceutical Product Development, Dubai Pharmacy College for Girls, Dubai, United Arab Emirates; 2 Pharmaceutics Department, Dubai Pharmacy College for Girls, Dubai, United Arab Emirates; 3 Department of Clinical Pharmacy and Pharmacotherapeutics, Dubai Pharmacy College, Dubai, United Arab Emirates; 4 Department of Pharmacognosy, Faculty of Pharmacy, Cairo University, Cairo, Egypt; 5 Institute of Polymers, Composite, and Biomaterials (IPCB), National Research Council of Italy, Naples, Italy; Central University of Rajasthan, INDIA

## Abstract

**Aim:**

Formulation of Pomegranate Extracts (PE)-loaded sphingosomes as an antitumor therapy for the intravenous and passive targeted delivery to various tumor types, especially that of the breast, colon, and uterus; to increase the therapeutic activity and decrease the adverse effects profile.

**Methods:**

The pericarp and seeds’ juice of *Punica granatum* were each extracted using D.W. and ethanol. Phytochemical investigation of all extracts was carried out including total phenolics, flavonoids, and anthocyanins contents, the antioxidant activity, as well as HPLC analysis of phenolics and flavonoids. The antitumor potential of all extracts was also tested utilizing three cell lines: MCF-7, HeLa, and HCT116. The candidate extract was chosen for the formulation phase and was entrapped into the sphingosomes using the thin-film hydration method and employing three different PE: lipids weight ratios. The synthesized formulations were characterized for their size, morphological features, zeta potential, entrapment efficiency, and *in vitro* drug release and kinetics modeling studies. The optimized formula was further analyzed by FTIR spectroscopy and electron microscopy. The antitumor activity of F2 was also investigated using the same cancer cell lines compared to the plant extract.

**Results:**

The highest phenolics, flavonoids, and anthocyanins contents were observed in the ethanolic pericarps extract (EPE), followed by the ethanolic seeds extract (ESE). Consequently, EPE showed a higher antitumor activity hence it was selected for the formulation phase. PE-loaded sphingosomes formula (F2) was selected for having the highest EE% (71.64%), and a sustained release profile with the highest *in vitro* release (42.5±9.44%). By employing the DDSolver, the Weibull model was found the most suitable to describe the PE release kinetics compared to other models. The release mechanism was found to follow Fickian diffusion. Simulated pharmacokinetic parameters have portrayed F2 as the candidate formula, with the highest AUC (536.095) and slowest MDT (0.642 h). In addition, F2 exhibited a significant (p>0.05) stronger and prolonged anticancer effect against MCF-7, HeLa, and HCT116 cell lines at all concentrations tested compared to the free extract.

**Conclusion:**

The results proved that sphingosomes are an effective delivery system, improving pharmacological efficacy and reducing serious side effects of anticancer medications and natural products.

## 1. Introduction

**Fig 1:**
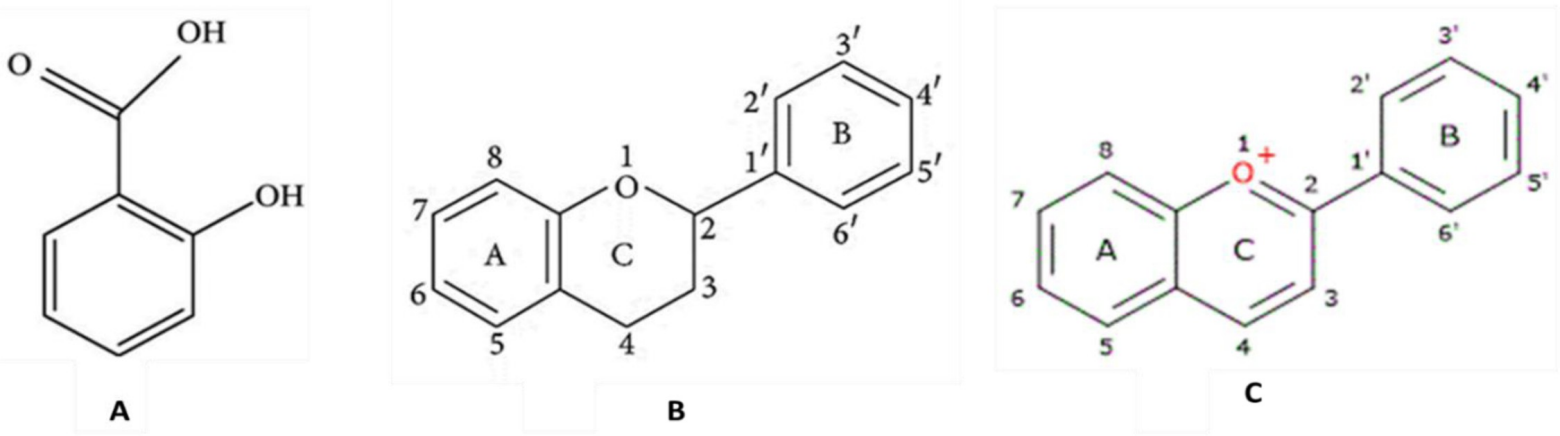
Basic chemical structures of A) Phenolic acids; B) Flavonoids; and C) Anthocyanins.

Pomegranate (*Punica granatum L*.) is a high-nutrient, phytochemical-rich fruit belonging to the family *Lythraceae*. The majority of it is grown in the Middle East, Asia, India, North and Tropical Africa, and Latin America [1]. In recent years, pomegranate research has gained the attention of researchers due to its nutritional value and therapeutic potential. There have been several studies that highlighted the functional properties of pomegranate and its extracts [2]. The main phytochemicals behind such activities are thought to be flavonoids, phenolic acids, antioxidants, and anthocyanins, which exist in all parts of the fruit, including the peel, arils, and seeds [3]. The chemical structure of these phytochemicals is presented in Fig 1.

According to the literature, the aforementioned phytochemical compounds can function as potent antioxidants, anticarcinogenic, antimutagenic, anti-diabetic, anti-ulcer, and anti-microbial agents [4]. They are also capable of preventing the onset and progression of a wide range of noncommunicable diseases, including cardiovascular, metabolic, and neurodegenerative diseases, as well as certain types of cancer [5]. Studies have demonstrated an enhanced function when using different pomegranate extracts concomitantly, especially in suppressing cell growth in multiple *in vitro* models, including uterine HeLa, human prostate DU145, human breast MCF-7, and PC-3 cancer cell lines. This was attributed to the active compounds in pomegranate extracts having an additive and synergistic effect, making them more powerful than any of these components alone [6].

On the other hand, the development of novel drug delivery systems has received a lot of attention over the past few decades Such systems have the advantage of releasing APIs at a predetermined rate throughout treatment, as well as selectively targeting the release to diseased tissues or organs, thereby minimizing adverse effects [7]. Various innovative pharmaceutical carriers are presented for modifying drug release and targeting. These include micro/macro molecules, particulate systems, and polymeric micelles, to list some [8]. Vesicular drug delivery systems (VDDSs), among these, are widely well-known such as liposomes, niosomes, transferosomes, phytosomes, and sphingosomes. They have the additive advantage of enhancing the therapeutic window, stability, and solubility of drug molecules [9].

Sphingosomes are amphiphilic concentric vesicular delivery systems formed of a sphingolipid bilayer enclosing an aqueous volume and having an acidic internal pH [10]. These vesicles are made up of sphingolipid (SL) and cholesterol (Chol) that occur in a proportion range of 75:25 to 30:50 molar ratios, with a 55:45 SL/Chol being the most desirable [9]. Several characteristics set sphingosomes apart from phospholipid-based VDDSs, making them more favorable. In terms of stability, the amide and ether links in the sphingosomes’ backbone make them more resistant to hydrolysis [11], and they are also less susceptible to rancidity and aggregation [12]. Moreover, sphingosomes are characterized by a longer biological half-life owing to the large hydrophilic group that shields their surface’s negative charge and limits RES clearance. As a result, the concentration of the loaded therapeutic agent increases at the site of action. Additionally, the enhanced stability and efficiency of these vesicles and their nanosize have allowed for their use in targeted drug delivery to tissues or organs for tumor and cancer therapy, immunology and gene therapy, enzyme delivery, as well as diagnostic and cosmetic purposes. It is worth noting, however, that the majority of the sphingosomes’ therapeutic applications that have advanced to the pre-clinical and clinical phases are in the field of cancer [13].

Based on the foregoing context, and since pomegranate extracts have shown great promise in cancer therapy but have yet to be studied using sphingosomal formulations, this study aimed at standardizing the ethanolic and aqueous extracts of the pericarp and the juicy seeds of Spanish pomegranate fruits, *Punica granatum L*., family *Punicaceae*, compare their anticancer activities using MCF-7, HeLa, and HCT116 cancer cell line and then load the candidate extract into three sphingosomal formulations that were prepared, characterized, and compared in terms of the *in vitro* release profile and cytotoxic activity. The primary goal is to achieve a controlled release of the extract’s phytochemicals while also increasing its antitumor activity against the tested cell lines.

## 2. Materials and methods

### 2.1 Plant materials

Whole fruits of *Punica granatum* L., F. Lythraceae of Spanish origin were bought in October 2020 from the local market in Dubai, United Arab Emirates. The fruits were carefully identified and authenticated by Prof. Naglaa Gamil Shehab “Department of Pharmacognosy, Cairo University, Cairo, Egypt”, “Department of Clinical Pharmacy and Pharmacotherapeutics, Dubai Pharmacy College for Girls, United Arab Emirates”. Two parts of the fruits were used; the pericarps, which were peeled, air-dried, and ground into a fine powder, along with the separated pomegranate seeds’ fleshy part. All were preserved for further studies.

### 2.2 Cancer cell lines used

The cell lines of the human breast carcinoma (MCF-7), the human colorectal carcinoma (HCT116), and the human cervical carcinoma (HeLa cells) were obtained from the National Cancer Institute, Cairo University, Cairo, Egypt.

### 2.3 Chemicals

All solvents were of analytical grade and obtained from Fisher Scientific (UK), while quercetin and gallic acid were purchased from Sigma-Aldrich (Germany). Sphingomyelin (SM) was obtained from bovine brain (MW: 731.078 g/mol) (Santa Cruz Biotechnology, USA) while Chol (MW: 386.66 g/mol) was obtained from (ALPHA CHEMIKA, India).

### 2.4 Preparation of pomegranate extracts

The fresh fruit’s pericarps and seeds of *Punica granatum L* were separated and weighed (1.49 kg and 1.3 kg, respectively). The pericarps were then air-dried and powdered. The powder was divided equally and was macerated exhaustively by cold maceration using absolute ethanol (3L) and distilled water (1L), while the seeds were mixed separately with absolute ethanol (2L) and distilled water (1L) using a blender. All extracts were filtered. The ethanolic extracts were separately evaporated at 60°C under reduced pressure, using a rotary evaporator.

On the other hand, the aqueous extracts were freeze-dried at -46 C°, under 1 pa, and for 36 hrs using a lyophilizer (BK-FD10P, Biobase, China). All extracts were stored in the fridge at 4°C for further analysis.

### 2.5 Standardization of the pomegranate extracts

#### 2.5.1 Colorimetric monitoring of phenolic and flavonoid contents in different extracting solvents

Colorimetric measurements of total flavonoid and phenolic contents of the pericarps and the fleshy part of the seeds for both ethanolic and aqueous extracts were determined spectrophotometrically using a UV-visible spectrophotometer (UV1800, Shimadzu, Japan) to assess the efficiency of the extracting solvents. All experiments were carried out in triplicate. The total phenolic content was estimated by using the Folin-Ciocalteu reagent. according to the procedure published by Singleton and Rossi [14] and modified by Unir *et al*. [15]. The results were represented as g/100g gallic acid equivalents based on the dry weight of the plant material; the calibration curve was established using serial dilutions of gallic acid (10, 20, 30, 40, and 50 μg/mL). The absorbance was measured against a reagent blank at λ_max_ 750 nm.

Alternatively, the total flavonoid content of the extracts was determined, spectrophotometrically, following the aluminum chloride method in which quercetin was used as a standard. The calibration curve was established using serial dilutions of quercetin (10, 20, 30, 40, and 50 μg/mL) according to the method described by Dewanto *et al*. [16]. The absorbance of the yielded yellow color is then spectrophotometrically measured at λ_max_ 510 nm.

#### 2.5.2 Determination of total anthocyanins content

Using spectrophotometric measures, the total amount of anthocyanins in each extract of *Punica granatum* was determined as described by Román *et al*. [17]. A volume of 2 mL of the extract’s solution was diluted up to 25 mL using a mixture (pH 1.0) of 0.2 M HCl (375 mL) and 0.2 M KCl (125 mL). Another fresh 2 mL volume of the extract’s solution was diluted up to 25 mL with a buffer solution (pH 4.5) made of 1 M HCl (240 mL), 1 M sodium acetate (400 mL), and H_2_O (360 mL). The absorbance of the two solutions was then measured at λmax 510 nm and anthocyanins concentration was calculated using *Eq 1*

$$C_{mg/L}=(Abs_{pHI}-Abs_{pH4.5})*484.82*1000/24825*DF$$ (1)

Where; (Abs _pH1_ –Abs _pH4.5_) is the difference of absorbance at 510 nm between pH 1.0 and pH 4.5 solutions,

484.82 is the molecular mass of cyanidin-3-glucoside chloride,

24825 is the molar absorptivity (ε) of cyanidin-3-glucoside chloride at 510 nm in the solution (pH 1.0 ±0.1), and DF is the dilution factor.

### 2.6 HPLC analysis of the pomegranate extracts

The composition of pomegranate extracts, both aqueous and ethanolic, was evaluated using RP-HPLC analysis by an Agilent 1100 HPLC system equipped with a C18-MS packed column (5 μm, 4.6 mm i.d. × 125 mm). The extracts were filtered through a 0.45 μm Millipore® syringe filter before being fed into the column, with an injection volume of 10 μL for both the tested samples and the standards. The analysis was conducted under conditions that allowed for the identification of phenolic acids or flavonoids [18]. Separation of phenolic acid compounds was done by applying a gradient mobile phase of two solvents, namely methanol and acetic acid in water (1:25, *v/v*) as described by Lin Y L *et al*. [19], and the phenolic components were measured at λ = 280 nm. Whereas, an isocratic flow of a binary mixture of methanol /water (50:50 *v/v*), adjusted to pH 2.8 with phosphoric acid at a flow rate of 1mL/min., was used for the determination of flavonoids [20] and the flavonoid components were measured at λ_max_ = 360 nm. The obtained chromatograms were analyzed using the Agilent ChemStation. Individual components were identified by comparing the retention times of unknown peaks to those of reference standards. All samples were performed in triplicates.

### 2.7 Antioxidant activity: DPPH radical scavenging assay

The antioxidant activity of the pomegranate extract in D.W. and absolute ethanol was determined through the radical scavenging capacity employing a stable DPPH radical. The assay was carried out in a 96-well microtiter plate utilizing the formerly reported modified technique by Shehab *et al*. [18]. The reaction mixtures were shaken vigorously before being incubated at 37°C in the dark for 30 min., and the absorbance was spectrophotometrically measured at λ_max_ 517 nm. The following formula was used to compute the percentage of the test samples that inhibit the DPPH radical:

$$Percentage\;Inhibition\;(\%)=]A_{0}-\left(A_{1}-A_{2}\right)\left[/A_{0}[\times 100\%\right.$$ (2)

Where; A_0_ is the absorbance of the control, A_1_ is the absorbance in the presence of the sample, and A_2_ is the absorbance of the sample under identical conditions as A_1_ with ethanol instead of DPPH solution. Ascorbic acid was used as a reference standard and samples were examined in triplicate.

### 2.8 *In vitro* cytotoxicity study of the pomegranate extracts by MTT colorimetric assay

This study has been approved by the Research Unit of Dubai Pharmacy College for Girls (Reference No. REC/Mpharma/PPD/2021/04) and all participants signed written informed consent before participating in this study. For this study, three different cancer cell lines were employed. These are human breast carcinoma (MCF-7), human colorectal carcinoma (HCT116), and human cervical carcinoma (HeLa cells) cell lines.

This investigation was carried out following the procedure described in the references [21, 22]. In brief, A 96-well tissue culture plate was inoculated with 1x10^5^ cells /mL (100 μL /well) and incubated at 37°C for 24 hrs to develop a complete monolayer sheet. The growth medium was then decanted after a confluent sheet of cells was formed which was washed twice with wash media. In the RPMI medium, which acted as a maintenance medium, two-fold dilutions of the tested sample were made with 2% serum. After that, a volume of 0.1 mL of each dilution was tested in different wells leaving three wells as control, receiving only maintenance medium. The 96-well tissue culture plate was then incubated at 37°C and examined. The cells were checked for any physical signs of toxicity, such as partial or complete loss of the monolayer sheet, rounding, shrinkage, or cell granulation. Following that, a 5mg/mL solution of MTT in PBS was prepared, and 20 μL of it was added to each well. The plate was then shaken at 150 rpm for 5 min. before being incubated at 37°C in a 5% CO_2_ atmosphere for 1–5 hrs to allow for MTT metabolism. The medium was then disposed and 200μL of DMSO was used for the solubilization of Formazan (MTT metabolic product). This was followed by agitating the tissue culture plate on a shaking table for 5 min. at 150 rpm to thoroughly mix the formazan into the solvent. Finally, the optical density, which is directly proportional to the viable cell number per well, was spectrophotometrically analyzed at 560 nm.

### 2.9 Preparation of PE-loaded sphingosomes

PE-loaded sphingosomes were prepared by the thin-film hydration method with modifications [23]. The specific weight of the SM and Cholesterol in a weight ratio of (55:45 *w/w*) was dissolved in 8 mL of chloroform. While the PE (40.2 mg) was dissolved in 2 mL of methanol. Both solutions were then mixed and placed in a round bottom flask. The mixture was evaporated at 65°C in a rotary evaporator (Laborota 4000 efficient, Heidolph, Germany) at 100 rpm under reduced pressure until a dry thin film was obtained on the walls of the flask. Then, 5 mL of PBS (pH 7.4) was added to the film, sonicated for 5 min., and left for 2 hrs for complete hydration. The resultant milky white sphingosomal dispersions of all the formulas were then lyophilized using a BK-FD10P freeze drier (Biobase, China). The system was cooled to—46 C°, under a vacuum pressure of 1pa for 36 hrs. The dried powdered samples were then stored at 4 ± 2°C and utilized for further investigations.

**Fig 2:**
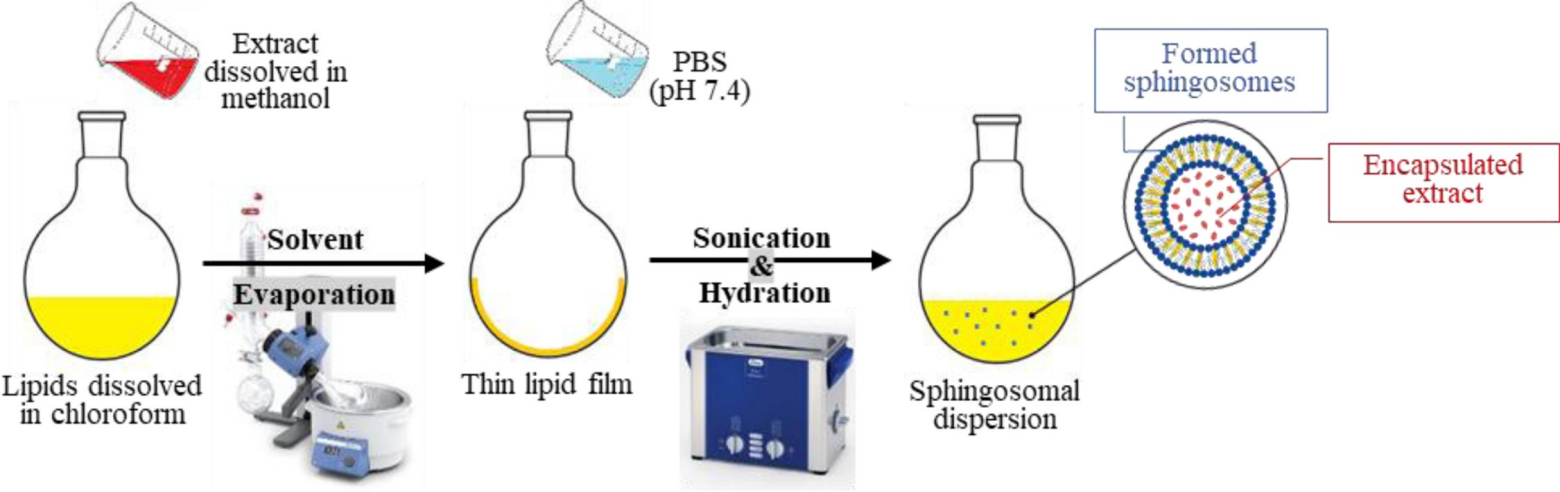
Preparation of PE-loaded sphingosomes by lipid hydration method.

The compositions of the prepared formulations are shown in Table 1 and the procedure of the preparation is summarized in Fig 2.

**Table 1:** Composition of PE-loaded sphingosomes formulations.

Batch Code	Ingredients					PE: lipids weight ratio (*w/w*)
	SM (mg)	Cholesterol (mg)	PE extract (mg)	Solvent (mL)	PBS (mL)	
Blank	40.2	17.39	0	Chloroform (10 mL)	5	N/A
F1	40.2	17.39	40.2	Chloroform (8 mL)	5	1:1
				+		
F2	80.4	34.79		Methanol (2 mL)		1:2
F3	120.6	52.19				1:3

### 2.10 Preparation of PE calibration curve

**Fig 3:**
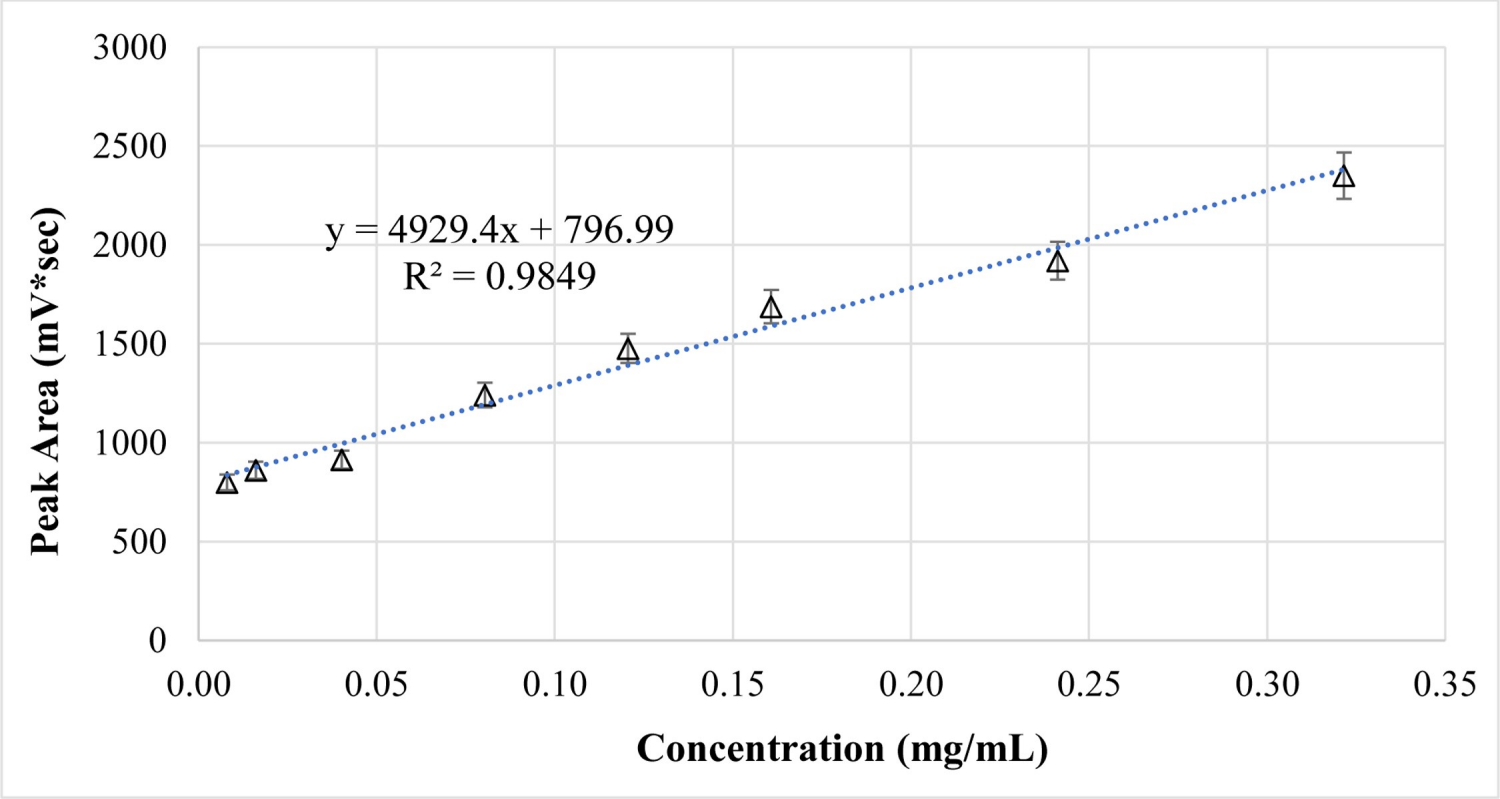
HPLC-calibration curve of the PE in PBS (pH 7.4). The points are given as mean ±SD (n = 3).

The calibration curve of the PE in PBS (pH 7.4) was prepared by making a series of dilutions (0.08, 0.16, 0.32, 0.4, 0.56, 0.64 mg/mL) from a stock solution of 0.8% *w/v* of PE in phosphate buffer pH 7.4 using the HPLC at λmax 275nm. The calibration curve was plotted, and linear regression analysis was performed using Microsoft® Excel sheet 2019; to determine the linear equation as well as the correlation coefficient (R^2^). The high R^2^ value suggested that the concentrations used in the calibration curve construction were convenient and followed Beer’s law. All results were plotted as mean ±SD (Fig 3).

### 2.11 Characterization of PE-loaded sphingosomes

#### 2.11.1 Morphological examination

All the prepared sphingosomal formulations were observed under an optical microscope fitted with a digital camera (Carl Zeiss, Germany). A drop of the sphingosomes dispersions was placed on a clean slide and examined at 40X magnification to detect the existence and the shape of the vesicles.

Furthermore, the morphological features, surface topography, and vesicle size of the selected sphingosomal formulation (F2) and its blank were investigated by SEM and TEM (ThermoScientific Apreo C, Czech Republic). A sample of each was placed on an aluminum specimen stub using conductive tape, followed by a 10 nm gold coating by a Sputter Coater (Q150TS, Quorum technology, UK) under reduced pressure. The samples were then subjected to SEM imaging. However, for TEM analysis, a sample of each of the lyophilized F2 and the blank formula were mixed in pure ethanol and vortexed for 5 min. and the liquid solution was deposited on a 200 mesh copper grid and analyzed using the TEM detector.

#### 2.11.2 Vesicle size, size distribution, and zeta potential

The vesicle size, polydispersity index (PDI), and zeta potential were determined by using the laser diffraction particle size analyzer (Litesizer 500, Anton Paar, Austria). A 0.1mL of the sphingosomal formulation was diluted with 10mL of PBS (pH 7.4), filtered with a Millipore membrane filter, and placed into a disposable cell for particle size and PDI measurement. On the other hand, an omega cell was used to determine the zeta potential [24]. All the measurements were represented as mean values ±SD (n = 3).

#### 2.11.3 Determination of entrapment efficiency (EE%)

The EE% was measured by placing 1 mL of the sphingosomal dispersion in the Eppendorf^®^ tubes. This was then placed in a cooling micro-centrifuge (3520, KUBOTA, Japan) and centrifuged at 15000 rpm for 30 min. at 4°C. The supernatant containing the unentrapped drug (free drug) was collected, diluted with PBS pH 7.4, and analyzed by UV-visible spectrophotometer at λ_max_ 275 nm. The amount of free drug was calculated by referring to the PE calibration curve. The difference between the initial and free drug amounts was used to determine the amount of the entrapped drug. The EE% was then calculated using *Eq 3*:

EE%=(Entrappeddrugamount/totaldrugamount)×100 (3)

### 2.12 *In vitro* drug release study

The *in vitro* drug release of PE from the prepared sphingosomes was studied using the dialysis method. This was started by soaking the dialysis cassettes (10K MWCO, Thermo Fisher Scientific, USA) in the dissolution medium (PBS pH 7.4) for 2 min. before their use. One milliliter of the sphingosomal dispersion was then centrifuged in the cooling micro-centrifuge at 15000 rpm for 30 min. at 4°C. The separated PE sphingosomes were then dispersed in 1mL of D.W. and placed inside the dialysis cassette which was afterward immersed in 300 mL PBS pH 7.4. The temperature was adjusted to 37 ±0.5°C and the stirring rate to 100 rpm. Samples of 3mL were collected at predetermined time intervals (1, 2, 3, 4, 5, 6, 7, 8, 9, 10, 11, and 12 hrs) with the replacement of the same volume of fresh medium (PBS pH 7.4) to maintain the sink conditions [23]. The amount of drug released with time was analyzed using the HPLC system (1525, Waters, Singapore) at λ_max_ 275 nm as per the PE calibration curve in PBS pH 7.4.

#### 2.12.1 Drug release kinetics modeling

Data from the *in vitro* release study were analyzed using the Microsoft® Excel add-in program, DDSolver® 2010, to determine the kinetic parameters of the phenolic compounds released from the sphingosomes formulations using several kinetic models [25], as displayed in Table 2.

**Table 2:** Applied kinetic models for the *in vitro* release data of PE-loaded sphingosomes.

Kinetic Model	Equation
**Zero-order**	*C*_*t*_ *= C*_*0*_ *+K*_*0*_ *t*
	Where: C_t_ is the amount of drug released at time t, C_0_ is the initial concentration of drug at time t = 0, and K_0_ is the zero-order rate constant.
**First-order**	*log C = log C*_*0*_ *-K*_*1*_ *t / 2*.*303*
	Where: K_1_ is the first-order rate equation expressed in time^-1^ or per hour, C_0_ is the initial concentration of the drug, and C is the percent of drug remaining at time t.
**Higuchi**	*Q = K*_*H*_ *× t*^*1/2*^
	Where: K_H_ is the Higuchi dissolution constant, and *Q* is the amount of drug released at time t.
**Korsmeyer-Peppas**	*Mt /M∞ = K*_*kp*_ *t*^*n*^
	Where: Mt/M∞ is a fraction of the drug released at time t, Mt is the amount of drug released in time t, M∞ is the amount of drug released after time ∞, n is the diffusional exponent or drug release exponent, and K_kp_ is the Korsmeyer release rate constant.
**Hopfenberg**	*Mt / M∞ = 1- [1- k*_*0*_ *t / CL a]* ^*n*^
	Where k_0_ is the zero-order rate constant describing the surface erosion process, CL is the initial drug loading throughout the system, and (a) is the system’s half thickness.
**Baker-Lonsdale**	*f = 3/2 [1- (1-Mt/Mα)*^*2/3*^ *]—Mt / Mα = Kt*
	Where Mt / Mα is the fraction of drug released at time t.
**Weibull**	m = 1-exp [— {(t–Ti )^b^ } / a]
	Where: (a) is the scale parameter defining the time scale of the process, (Ti) is the location parameter, represents the lag time before the onset of the dissolution or release process, and (b) is the shape parameter that describes the shape of dissolution curve progression.

Based on the correlation coefficient (R^2^) obtained from the linear regression analysis, the model that demonstrated the best fitting of the data and best represented the phenolic compounds release mechanism from sphingosomes was chosen. In addition, the DDSolver® 2010 program was used to perform a simulated pharmacokinetic analysis for all prepared formulas.

### 2.13 FTIR spectroscopy study

FTIR spectroscopy was used to test the chemical compatibility of the PE with the components of the formed sphingosomes and detect any possible physicochemical interaction between them. This was accomplished by making samples of the PE, Sphingomylien, and producing sphingosomal formulas (F1, F2, and F3), then using an FTIR spectrophotometer to scan these samples individually in a wavenumber range of 4000 to 400 cm^-1^ and at a resolution of 4 cm^-1^ (IRAffinity-1S, Shimadzu, Japan).

### 2.14 Statistical analysis

All results were expressed as mean values ±SD (n = 3). One-way ANOVA analysis was employed to determine statistically significant differences at an alpha *p* level of 0.05. The statistical analysis was performed using IBM SPSS^®^ statistics software, version 28.0.1.0.

## 3. Results and discussion

### 3.1 Yield of extractives

Extraction of the pomegranate seeds and powdered pericarps with absolute ethanol and D.W. rendered dark red residues in a yield of 43.19g ethanolic and 3.23g aqueous seeds extracts in comparison to 164.9g ethanolic and 6.51g aqueous pericarp extracts.

### 3.2 Standardization of the pomegranate extracts

#### a) Colorimetric analysis of phenolic and flavonoid contents in the extracting solvents

The total flavonoid and phenolic contents of the ethanolic and the aqueous extracts of the juicy part of the seeds and the pericarps of *Punica granatum* were investigated spectrophotometrically. Both the ethanolic and the aqueous extracts of the pericarps showed the highest flavonoid and phenolic contents compared to the extracts of the seeds (1.2% and 0.96% flavonoid content, respectively) and (1.75% and 1.68%, phenolic content respectively).

On the contrary, ethanolic and aqueous seeds’ extracts had flavonoid contents of (0.09% and 0.08%, respectively) compared to the shared phenolic content (both extracts 0.14%). Similar results were reported by Barchan *et al*., where the pericarp extract of *Punica granatum* was shown to have the highest overall phenolic and flavonoid concentrations, compared to other parts of the fruit [26]. On the other hand, increasing the polarity of the solvent didn’t significantly (*p*> 0.05) increase the concentration of flavonoids or phenolic acids in either the pericarp or the seeds extract.

#### b) Determination of total anthocyanin content

Spectrophotometric evaluation of the four *Punica granatum* extracts at λmax 510nm revealed anthocyanins content that ranged between 2.32±0.17 mg/L and 115.96±7.94 mg/L.

**Fig 4:**
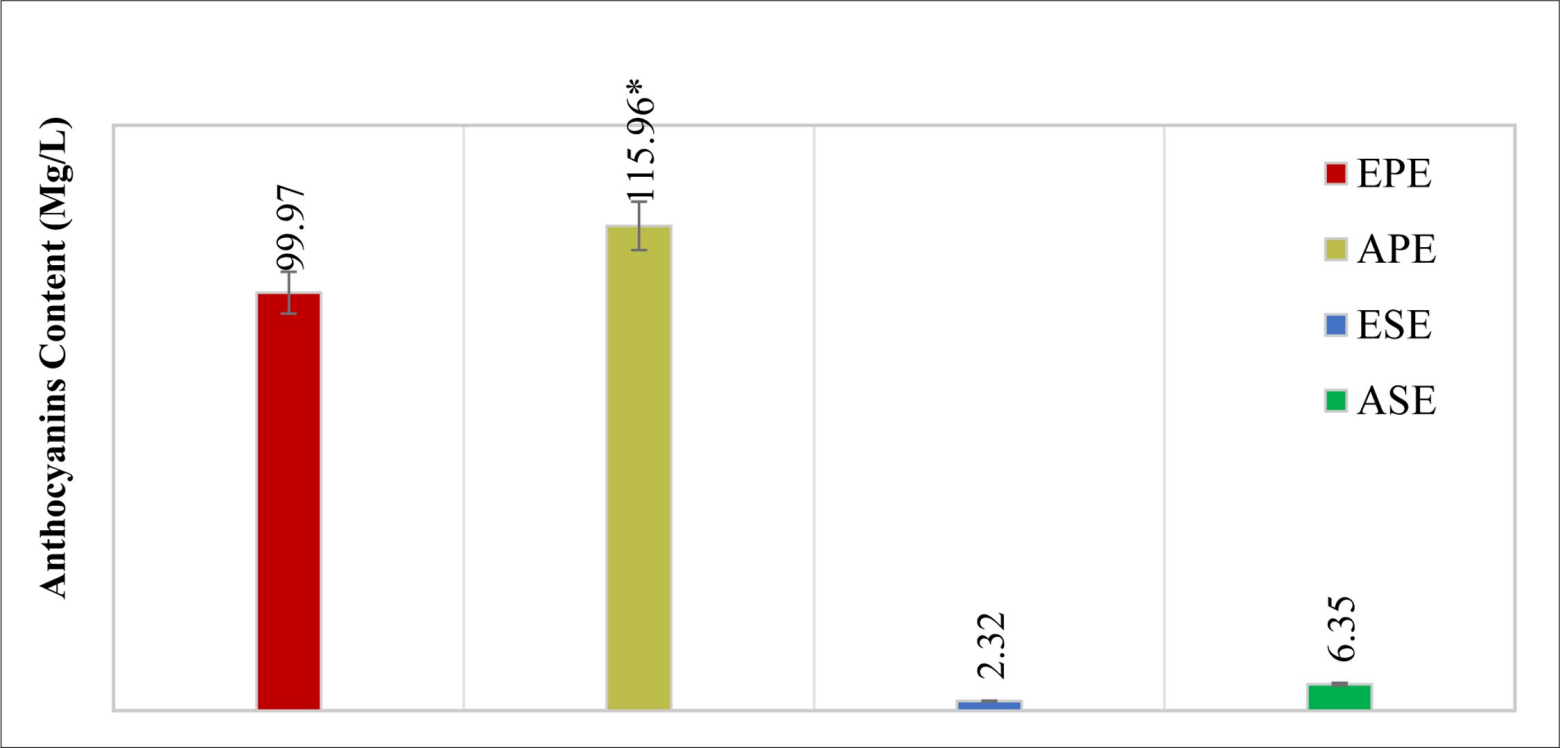
The anthocyanins content of the four extracts of *Punica granatum*. EPE: Ethanolic Pericarp Extract, APE: Aqueous Pericarp Extract, ESE: Ethanolic Seeds Extract, and ASE: Aqueous Seeds Extract.

Furthermore, statistical analysis of the results revealed a significant difference between the anthocyanins content in the pericarp extracts and that of the extracts of the seeds (*p*< 0.05). It was noted that the aqueous pericarp extract had the highest content of anthocyanins (115.96±7.94 mg/L), followed by the ethanolic pericarp extract (99.97±0.17 mg/L), the aqueous seeds extract (6.35±0.69 mg/L), and the ethanolic seeds extract (2.32±0.17mg/L), respectively (Fig 4).

On the other hand, increasing the polarity of the extraction solvents (from absolute ethanol to D.W.) increased the anthocyanin contents in both the pericarp and the extracts of the seeds, however, this impact was statistically insignificant (*p*> 0.055). Our findings follow that of a study by Ali *et al*., where authors investigated the anthocyanins content in the peel, flesh, seeds, and whole fruit of *Punica granatum*, and concluded that the peel contains the highest quantity of anthocyanins [27].

### 3.3 RP-HPLC analysis of *Punica granatum* extracts

**Fig 5:**
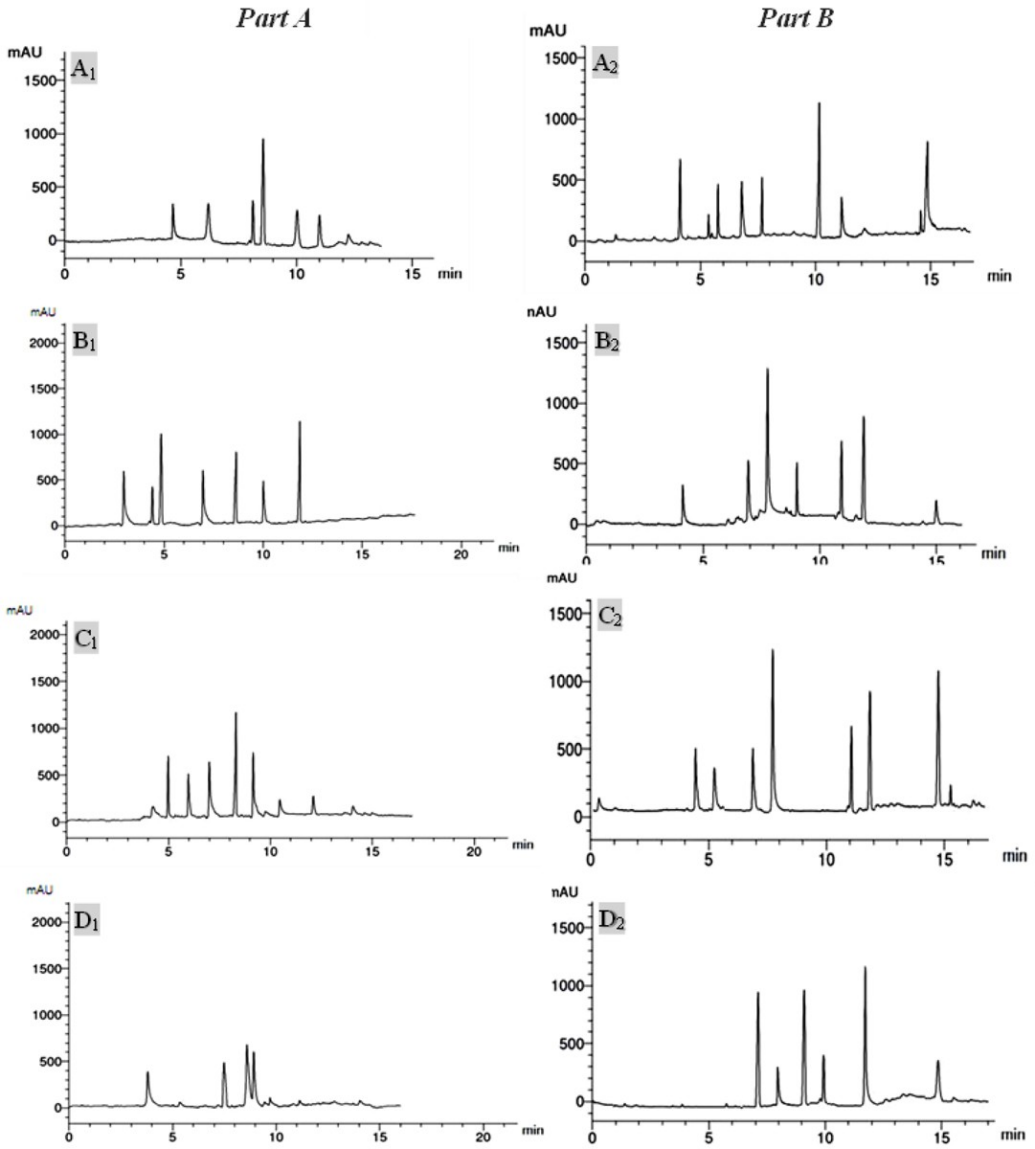
RP-HPLC chromatograms of ethanolic pericarp extracts (A1, A2), aqueous pericarp extracts (B1, B2), ethanolic seeds extracts (C1, C2), and aqueous seeds extracts (D1, D2). Where part A demonstrates the analysis of phenolic acids and part B shows the analysis of flavonoid components.

RP-HPLC evaluation of the extracts of *Punica granatum* enabled the identification and quantitation of a variety of flavonoid and phenolic compounds, as shown in Tables 3, 4 and Fig 5.

**Table 3:** Phenolic compounds identified by RP-HPLC analysis of the ethanolic and aqueous extracts of *punica granatum* (λ = 280 nm).

Retention time (min.)	Identified phenolic compounds	Concentration (μg/mL)			
		Pericarp		Seeds	
		*Ethanolic Ext*.	*Aq. Ext*.	*Ethanolic Ext*.	*Aq. Ext*.
3	Catechol	**-**	**-**	**-**	9.14
4.267	Caffeic acid	7.41	0.44	4.66	4.33
5	Ferulic acid	5.17	7.66	**-**	14.08
6	O-Coumaric acid	**-**	5.97	**-**	**-**
	P-Coumaric acid	6.33	**-**	**-**	**-**
7.167	Gallic acid	4.98	7.47	5.87	7.68
7.89	Chlorogenic acid	**-**	11.69	**-**	**-**
8.5	Syringenic acid	5.12	**-**	9.67	8.69
8.8	Pyrogallol acid	13.63	**-**	**-**	**-**
9.1	P-OH benzoic acid	**-**	8.43	7.51	**-**
10	Cinnamic acid	**-**	**-**	0.078	7.65
11	Salicylic acid	**-**	0.78	**-**	**-**
12.1	Ellagic acid	**-**	0.69	**-**	17.36
14.2	Protocatechuic acid	**-**	0.13	**-**	**-**

Ethanolic Ext: Ethanolic extract, Aq. Ext: Aqueous extract.

**Table 4:** Flavonoid compounds identified by RP-HPLC analysis of the ethanolic and aqueous extracts of *punica granatum* (λ = 360 nm).

Retention time (min.)	Identified Flavonoid compounds	Concentration (μg/mL)			
		Pericarp		Seeds	
		*Ethanolic Ext*.	*Aq. Ext*.	*Ethanolic Ext*.	*Aq. Ext*.
4.3	Rutin	16.25	7.13	**-**	7.14
5.25	Naringin	5.66	6.19	**-**	
6	Isorhamnetin	10.23	**-**	**-**	**-**
6.95	Quercetin	9.66	8.47	15.36	9.56
7.97	Kaempferol	11.43	20.47	6.15	22.17
9	Luteolin	**-**	**-**	14.66	8.15
10	Hesperidin	22.15	**-**	8.12	**-**
11.033	7-OH flavone	8.14	14.16	**-**	12.02
11.978	Catechin	1.13	11.78	20.56	16.11
14.6	Genistein	3.52	**-**	**-**	**-**
14.975	Chrysoeriol	15.04	17.44	4.21	7.66
15.2	Myricetin	**-**	2.25	**-**	**-**

Ethanolic Ext: Ethanolic extract, Aq. Ext: Aqueous extract.

Six phenolic compounds were detected in the ethanolic pericarp extract in λmax 280nm, in comparison to 9 compounds in the aqueous pericarp extract. On the other hand, the number of phenolic acids identified in ethanolic seed extract was 5, whereas that of aqueous seed extract was 7 phenolic compounds.

Caffeic and gallic acids were detected in all investigated extracts in different concentrations. The major phenolic compounds in the ethanolic pericarp extract were pyrogallol acid followed by caffeic acid (13.63 and 7.41 μg/mL respectively), while chlorogenic acid, P-OH benzoic acid, and ferulic acids were the most plentiful in the aqueous pericarp extract (11.69, 8.43 and 7.47 μg/mL, respectively). Furthermore, syringenic acid and P-OH benzoic acid were the most abundant in the ethanolic seeds extract (9.67 and 7.51 μg/mL, respectively), in contrast to ellagic and ferulic acids that were the amplest in the aqueous seeds extract (17.36 and 14.08 μg/mL, respectively). Setting the detector to 360 nm, on the contrary, helped to identify 10 flavonoid components in the ethanolic pericarps extract, 8 flavonoids in the aqueous pericarps extract, and 6 and 7 flavonoids in the ethanolic seeds extract and aqueous seeds extracts, respectively.

Quercetin, kaempferol, catechin, and chrysoeriol were detected in all extracts in different amounts. Hesperidin, rutin, and chrysoeriol were the major components in ethanolic pericarp extract (22.15, 16.25, and 15.04 μg/mL, respectively). While kaempferol, chrysoeriol, and 7-OH flavone were the major in aqueous pericarps extract (20.47, 17.44, and 14.16 μg/mL, respectively). On the other hand, catechin and quercetin constituted the majority of ethanolic seed extract (20.56 and 15.36 μg/mL), and kaempferol and catechin were the most abundant in aqueous seed extract (22.17 and 16.11 μg/mL) (Table 4).

### 3.4 Antioxidant activity

**Fig 6:**
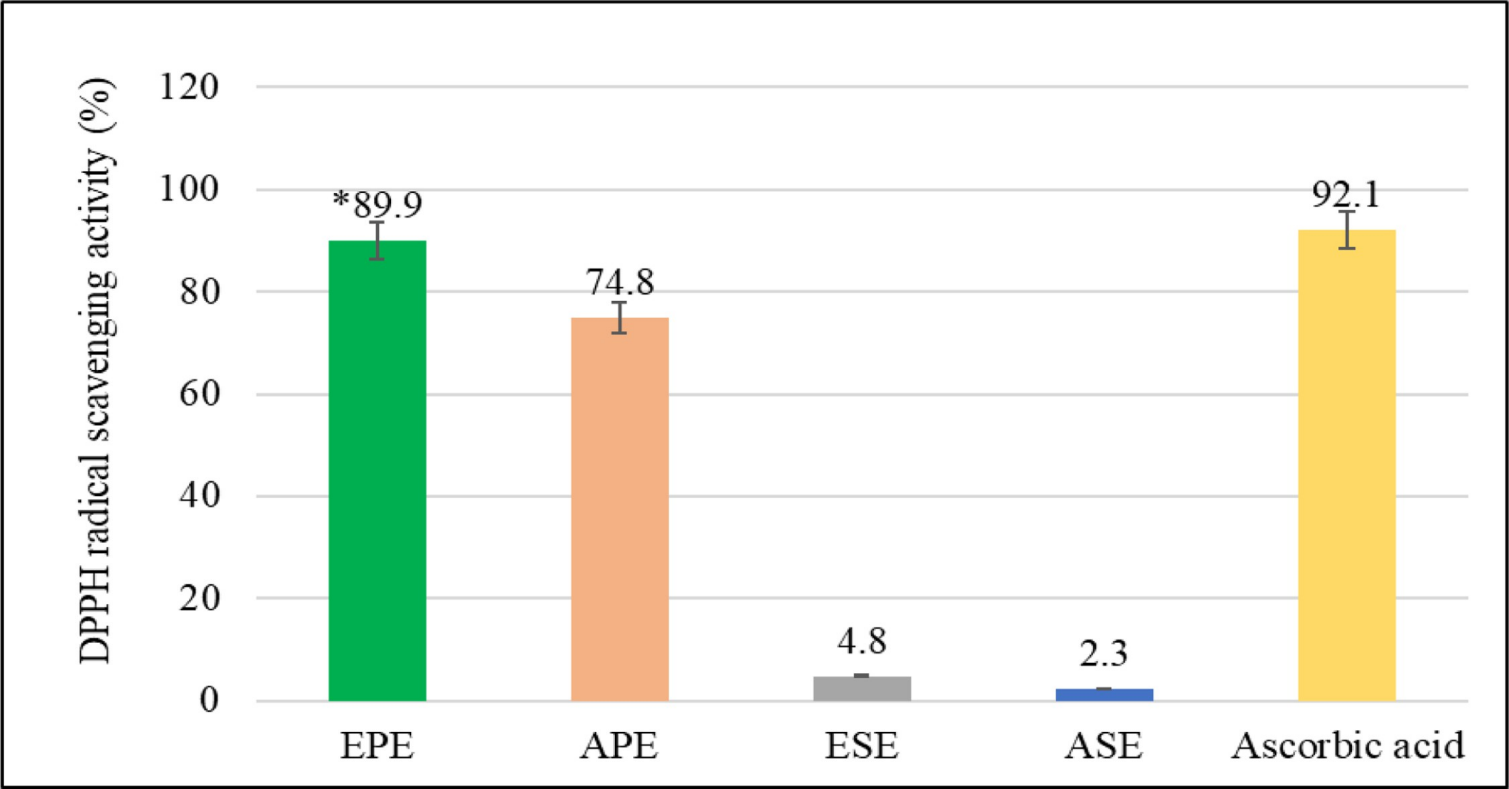
The antioxidant activity (%) of the four extracts of *Punica granatum*. EPE: Ethanolic Pericarp Extract, APE: Aqueous Pericarp Extract, ESE: Ethanolic Seeds Extract, and ASE: Aqueous Seeds Extract.

The stable free radical 2,2-diphenylpicrylhydrazyl (DPPH) displays a distinct purple color measured spectrophotometrically at λmax 517nm. When a plant extract with antioxidant activity is added to the DPPH assay solution, it donates a hydrogen atom that scavenges the free radical, changing the color of the solution to a yellowish hue, and leading to a reduction in the absorbance. The DPPH free radical scavenging activity is regarded as an *in vitro* screening for probable *in vivo* antioxidant capacities. The four extracts were tested for their antioxidant activities compared to ascorbic acid as a reference standard, and the results are presented in Fig 6.

The ethanolic pericarp extract was found to have significantly (*p*< 0.05) the highest antioxidant activity (89.9%), followed by the aqueous pericarp extract (74.8%). On the other hand, the ethanolic and aqueous seed extracts displayed the lowest antioxidant potentials, at 4.8% and 2.3%, respectively.

Similar findings were seen in a study by Derakhshan *et al*., in which the pericarp extract of *Punica granatum* outperformed the seeds and juice extracts in terms of antioxidant activity [28]. This might be due to the presence of phenolic acids, flavonoids, and anthocyanins in much higher amounts in the pericarp extracts, as reported previously.

### 3.5 Preparation of PE-loaded sphingosomes

The method employed for the preparation of the sphingosomes was found to be successful in the initial trials and consistent with previous research works [29]. The conditions for the preparation of the sphingosomes formulations were optimized by using solvents: chloroform (8 mL) and methanol (2 mL); hydration medium: 5mL of PBS pH 7.4; hydration time: 2 hrs; and hydration temperature:20°C.

### 3.6 Characterization of PE-loaded sphingosomes

#### 3.6.1 Optical microscope examination

**Fig 7:**
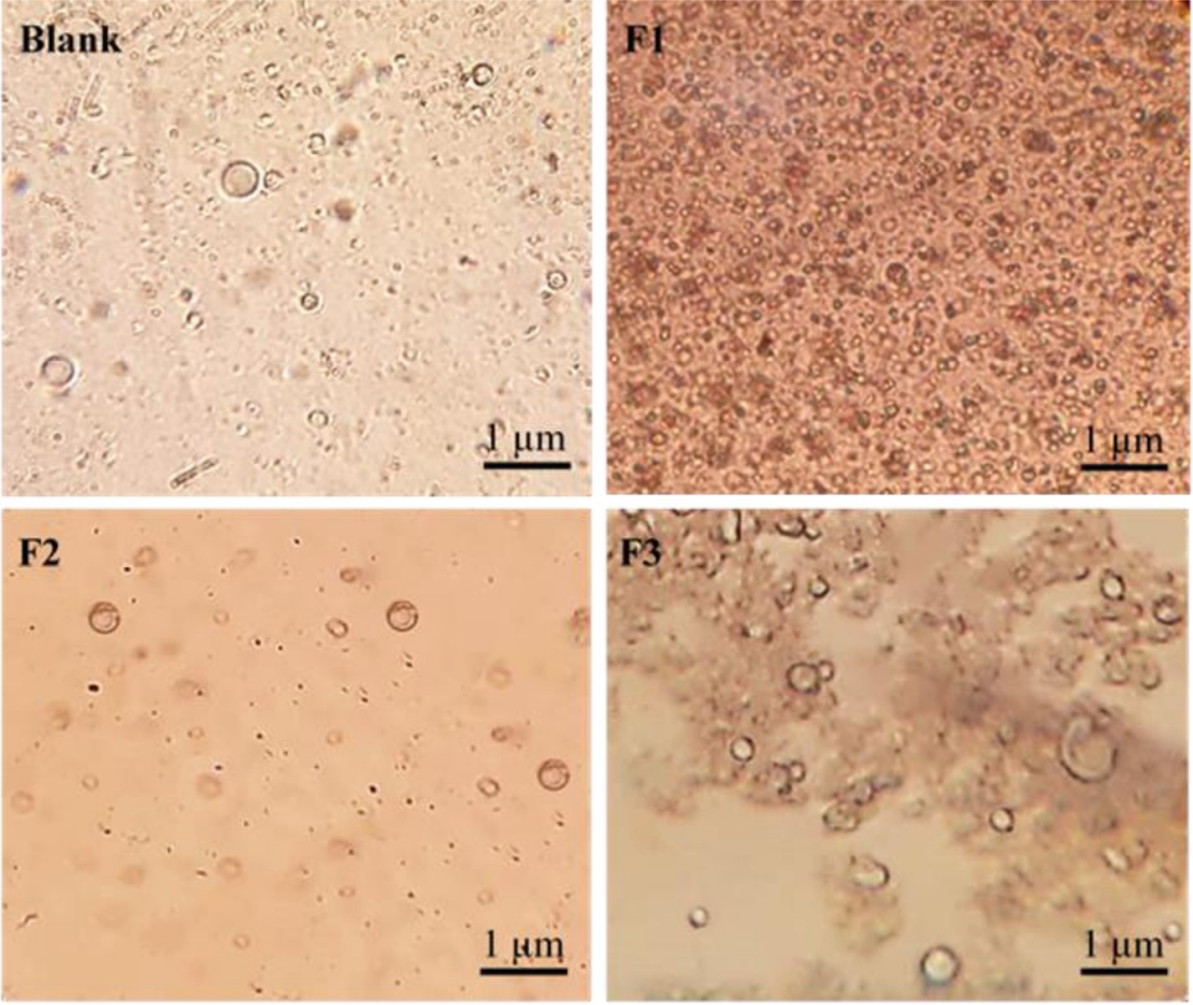
Micrographs of PE-loaded sphingosomes (F1-F3), and the blank under the optical microscope (40X).

Using the optical microscope, the existence of the sphingosomes vesicles was investigated and confirmed. The microscopical pictures revealed the formation of the sphingosomal vesicles (F1-F3). In addition, it was noted that the vesicles were spherical, distinct intact entities, and were abundant in the field specifically in batch F1 (Fig 7).

#### 3.6.2 Vesicle size, size distribution, and zeta potential

The size range of the prepared sphingosomes was from 93.2±4.49nm—182.7±18.3nm (Table 5). This nanosize was obtained by employing successive ultrasonication and extrusion to the formulations. Also, this finding was consistent with the observations of the SEM (section 3.6.8).

**Table 5:** Vesicle size, PDI, zeta potential, EE%, and Q12 of PE-loaded sphingosomes and the blank formula.

Formula	PE: Lipids (*w/w*)	Particle size (nm)	PDI (%)	Zeta Potential (mV)	EE (%)	Q12 (%)
**Blank**	0:1	93.2±4.49	27.1±3.4	-12.0±0.31	….	….
**F1**	1:1	101.1±13.5	15.2±4.3	-24.9±0.5	47.18±1.42	19.6±1.38
**F2**	1:2	126.0±13.05	13.8±3.2	-13.6±0.47	71.64±0.74	42.5±9.44
**F3**	1:3	*182.7±18.3	10.2±5.2	-11.1±0.79	57.88±1.81	36.8±6.70

* Significant results at *p*< 0.05. Results are presented as mean±SD (n = 3).

Ultrasonication and extrusion are among many techniques that have been used in previous studies to reduce the size of vesicular drug delivery systems [25]. Other techniques like homogenization and freeze-thaw sonication also proved a success in downsizing the liposomal vesicles by various percentages [30]. In addition, the results indicated that the vesicle size was affected by the weight ratio of PE to lipids in all formulations. F3 had the greatest size compared to the other two formulas and the blank vesicles. Moreover, the size of the loaded vesicles is significantly (*p*< 0.05) larger than the blank formula proving the loading of the plant extract into the vesicles (Table 6).

**Table 6:** *In vitro* release kinetics data of pe-loaded sphingosomes formulations by DDSolver software.

Modeling		Formulation		Rate constant	R^2^ adjusted	AIC	MSC	n
Zero-order		F1		4.9476	0.3854	22.9873	-1.3421	
		F2		11.0056	0.9516	19.8506	1.8895	
		F3		9.2284	0.8591	22.3372	0.6265	
First-order		F1		0.0570	0.4379	22.6302	-1.2529	
		F2		0.1540	0.9836	15.5192	2.9724	
		F3		0.1220	0.9106	20.5187	1.0812	
Higuchi		F1		10.7433	0.7933	18.6286	-0.2525	
		F2		22.6734	0.9816	15.9921	2.8542	
		F3		19.3097	0.9915	11.0885	3.4387	
Korsmeyer-Peppas		F1		18.1734	0.9982	0.1353	4.3709	0.064
		F2		18.7277	0.9885	14.4849	3.2310	0.185
		F3		20.7649	0.9906	11.8787	3.2412	0.059
Hopfenberg		F1		0.0003	0.1564	24.6321	-1.7533	198.6357
		F2		0.0001	0.9754	17.5244	2.4711	1059.0606
		F3		0.0002	0.8658	22.5225	0.5802	552.1781
Baker-Lonsdale		F1		0.0022	0.8098	18.2957	-0.1692	
		F2		0.0109	0.9722	17.6389	2.4425	
		F3		0.0076	0.9941	9.6306	3.8032	
Weibull		F1	α =	4.249	0.99911	-3.5714	5.2975	
			β =	0.005				
			Ti =	1.000				
		F2	α =	1.884	1.0000	-251.8508	69.8149	
			β =	0.198				
			Ti =	0.994				
		F3	α =	1.9771	1.0000	-10.3200	8.7912	
			β =	0.0269				
			Ti =	1.0000				
		**F1**	**F2**		**F3**			
Simulated PK Parameters	AUC	243.486	*536.095		456.460			
	MDT (h)	1.683	*0.642		1.438			

*Significant results at *p*< 0.05.

Our findings indeed were in agreement with those reported by Shaker *et al*., 2017 who also prepared nano-sized vesicles without the use of surfactants and concluded that the size of the formed vesicles was directly increased with the lipids’ concentration used in their preparation [31].

The polydispersity index (PDI) is a measure of the size distribution of a sample. The PDI percentage of the prepared formulas has ranged between 10.2±5.2 and 27.1±3.4 (Table 5). These PDI values were within the accepted value of (< 30%) which is specific to delivery systems utilizing lipid-based carriers [32] and indicated the homogenous distribution of the sphingosomes throughout the formulation dispersions.

On the other hand, zeta potential (ζ), which is the difference in electric potential across the ionic layer surrounding a charged colloid ion in a dispersed system, was measured to assess the charge stability of vesicular systems. In general terms, values of zeta potential greater than ±30 mV are thought to confer sufficient repulsive force to achieve colloidal stability. Conversely, a small zeta potential value denotes aggregation and flocculation of vesicles, which renders the colloidal dispersion unstable [33]. Many factors affect the magnitude of zeta potential, these include the pH of the medium, ionic strength, concentration of excipients, and temperature [34]. In this study, the formed sphingosomes in all formulations were found to be negatively charged. The reason behind this is owing to the lipid content, SM, which contains a phosphocholine head group that renders it a zwitterionic lipid (IEP = 6). Therefore, SM at a pH of 7.4 (hydration medium, PBS) carries a negative charge [35]. Our results showed that the ζ values ranged between -11.1±0.79 mV and -24.9±0.5 mV for the prepared formulations (Table 5). It was noticed that as the ratio of PE to lipids increased, the value of zeta potential increased as well, and this rise in ζ values was statistically significant (*p*< 0.05). On the contrary, our results disagree with those obtained by Calvagno *et al*., 2007, who demonstrated that adjusting the lipid molar ratio during liposomal preparation had an insignificant effect on the zeta potential of liposomal formulations [36]. Although the ζ values were < ±30 mV for all prepared formulations, which poses a risk of vesicle sedimentation and kinetic instability, were able to overcome this problem by lyophilizing the sphingosomal dispersions after they were prepared to transform them into powder samples. The powdered formulations were then stored in a firmly sealed container in a dark and dry environment until re-dispersed in PBS pH 7.4 just before any analysis.

#### 3.6.3 Entrapment efficiency (EE%)

The high loading efficiency of spingosomes for drug molecules of a wide range of solubilities is caused by passive loading during vesicle formation, which traps hydrophilic drugs within the sphingosome’s aqueous core and hydrophobic drugs within small hydrophobic lipid bilayers [8]. According to our results, PE-loaded sphingosomes showed an EE% ranging from 47.18±1.42% to 71.64±0.74% (Table 5). The maximum drug loading was obtained with F2 (PE: lipids weight ratio of 1:2). The results indicated that increasing the PE: lipids weight ratio from 1:1 to 1:2 resulted in significantly higher EE% (from 47.18±1.42% to 71.64±0.74%) (*p*< 0.05). However, raising the ratio to 1:3 resulted in a substantial decrease in EE% from 71.64±0.74% to 57.88±1.81% (*p*< 0.05). This fluctuating behavior in F3 may be attributed to the hydrophilic nature of the encapsulated PE. Since sphingosomes are composed of a lipidic bilayer enclosing an aqueous core, in which PE was entrapped, increasing the lipids composition of such a vesicle may have caused repulsion of the water-soluble extract to the outside leading to a lower EE%. On the contrary, contradicting results were reported by Mao *et al*., who have synthesized vincristine-loaded sphingosomes in varied drug: lipids ratios and observed a continual rise in EE% despite higher lipids content [37].

#### 3.6.4 *In-vitro* drug release study

Based on the findings in Section 3.1, it was observed that phenolic compounds (λ_max_ = 275nm) were the most abundant in PE, and thus were the most attributable to its superior anticancer activity. As a result, we traced the phenolic compounds released from the produced PE-loaded sphingosomal formulations in PBS (pH 7.4) for 12 hours at λ = 275nm. Because of the sphingosomes’ passive tumor-targeting property, which is mostly related to their nano-size [38], various studies have employed them for the targeted delivery of anticancer drugs to tumor cells, while utilizing the intravenous route of administration in their animal studies [39, 40]. Therefore, a pH of 7.4, similar to that of blood, was employed in our investigation to obtain a sustained release profile from the prepared sphingosomal formulas.

**Fig 8:**
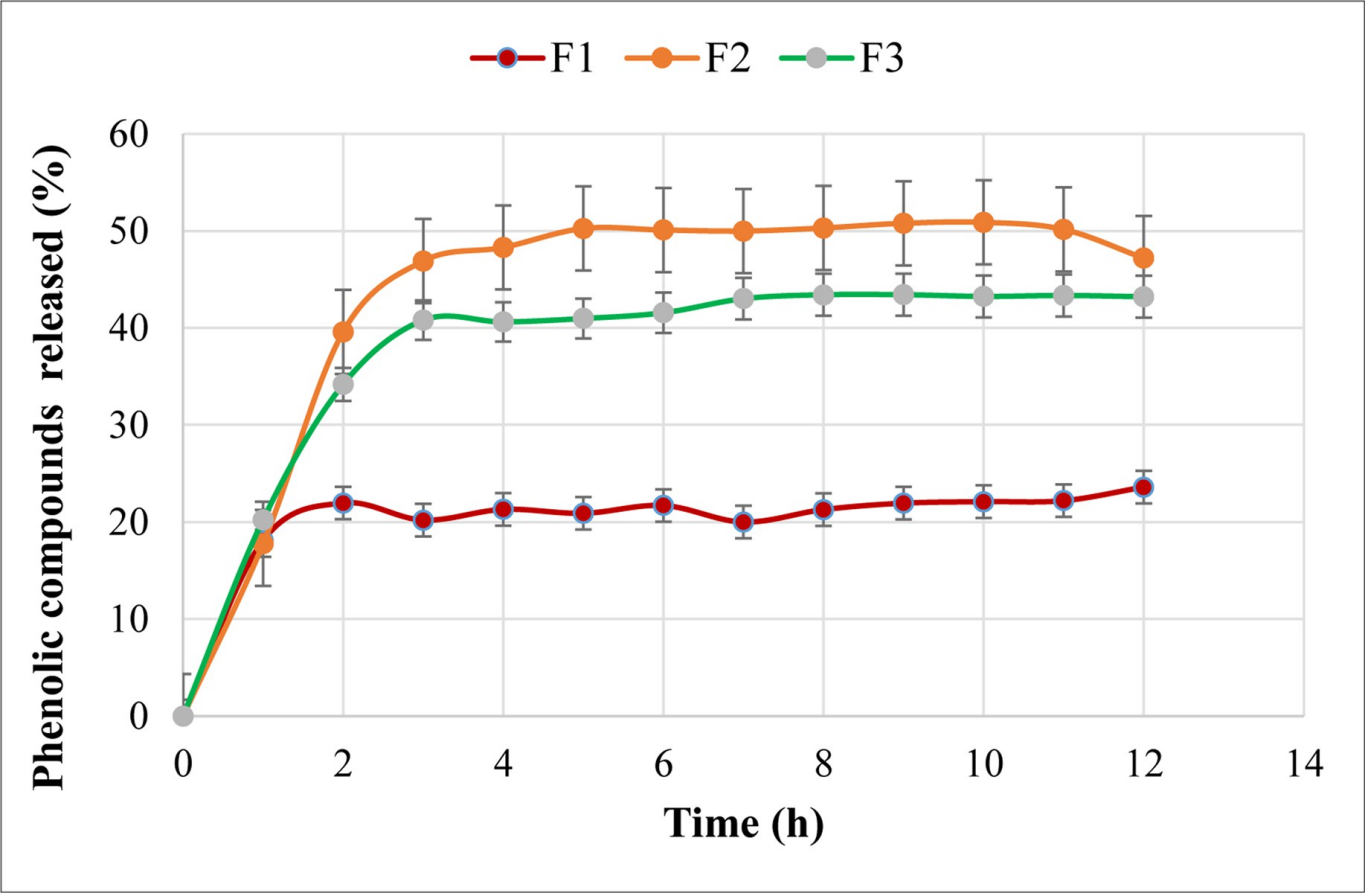
*In vitro* release of phenolic compounds from the prepared sphingosomes in 12 hrs run. The points are mean ±SD (n = 3).

F1-F3 showed a gradual increase in the phenolic compounds released into the dissolution medium during the first hour of the study (Fig 8).

This was followed by a sustained release pattern for F1, while the release from F3 and F2 continued to escalate until reaching a steady state on the third and fourth hours of the run, respectively. The mean phenolic compounds released after 12 hrs of the run (Q_12_) from F1, F2, and F3 were approximately 19.6%, 42.5%, and 36.8%, respectively, and the difference was highly significant (*p*< 0.01) (Table 5). Upon these findings, increasing the lipid content of the sphingosomes led to a commensurate substantial increase in the release of phenolic compounds between F1 and F2 (*p*< 0.05); however, further increasing the lipid content in F3 lowered the release by 19.2% compared to F2 (*p*< 0.05). These findings line with the observation of the EE% study and could be attributed to the lower PE loading to the F3 sphingosomes compared to F2 (36.8% and 42.5%, respectively). Similar results were obtained by Mao *et al*., who had also noticed a drug: lipids ratio-dependent behavior of the release profile of their prepared vincristine-loaded sphingosomes; the lower the ratio, the faster the release [37].

#### 3.6.5 Kinetics modeling and *in-vitro* release data analysis using DDSolver

The *in vitro* drug release data were fitted into various kinetic models including Zero-order, First-order, Higuchi, Korsmeyer-Peppas, Hopfenberg, Baker-Lonsdale, and Weibull. The linear regression was used to estimate the dissolution modeling by DDSolver® in line with the equations in Table 6.

For the selection of the model that offers the best fit of data, the adjusted correlation coefficient (R^2^_adjusted_) was used, where the model providing the highest (and closest to 1) value of R^2^_adjusted_ was selected. Based on this criterion, the Weibull model was found the most suitable model to describe the PE release kinetics from all the sphingosomal formulations (F1, F2, and F3) with R^2^_adjusted_ values of 0.9999, 1.0000, and 1.0000, respectively (Table 6).

According to the shape parameter (β) of this model, the release of the phenolic compounds was following Fickian diffusion, since the β value was ≤0.75 for all three formulations [41]. This was confirmed by the Korsmeyer-Peppas model, where the n value for F1, F2, and F3 was < 0.45, indicating the phenolic compounds released by Fickian diffusion as well [42]. One of the elements affecting the drug’s diffusion, according to Fick’s first law, is its dose or initial concentration, therefore, a rise in EE% corresponds to a higher initial concentration of the PE loaded into sphingosomes. As a result, there is a greater concentration gradient between the sphingosomes and the release medium, which leads to enhanced diffusion and consequently phenolic compounds’ release. For this reason, the highest release profile was exhibited by F2, which had the highest EE%, as reported earlier. Similar findings were reported by Shi *et al*. (2014) who also observed a proportional relationship between the drug’s initial concentration and its *in vitro* released amount [43].

Although the R^2^_adjusted_ was deemed the most appropriate parameter for comparing dissolution models, the results revealed a high degree of similarity between F1, F2, and F3. Therefore, other statistical criteria, such as the Akaike Information Criterion (AIC) and the Model Selection Criterion (MSC) were applied using the DDSolver software to validate the selection of the best model. For instance, a better fit of data into a kinetics model is indicated by lower AIC and higher MSC values. This was especially true for the Weibull model, which offered the highest MSC and lowest AIC values, indicating its superiority in fitting the experimental data of the three sphingosomal formulas (Table 6).

**Fig 9:**
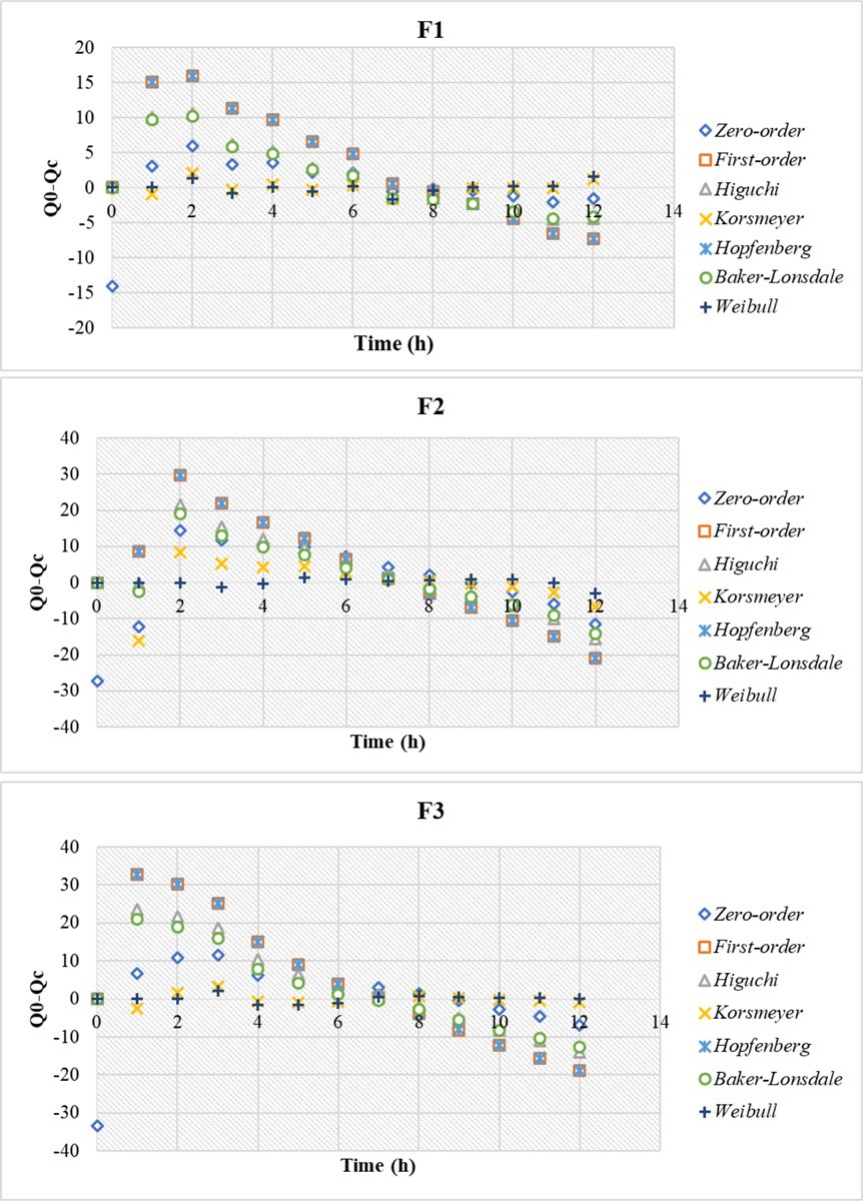
The correlation of residuals (Q0-Qc) vs. time for PE sphingosomal formulations F1, F2, and F3 by different dissolution models.

To further confirm the chosen model, the correlation of residuals (Q_0_-Q_c_) versus time was investigated for all the models employed. The Weibull model had the slightest deviation from the line for F1, F2, and F3, denoting it as the most appropriate kinetics model for describing the PE release from the prepared sphingosomes (Fig 9).

**Fig 10:**
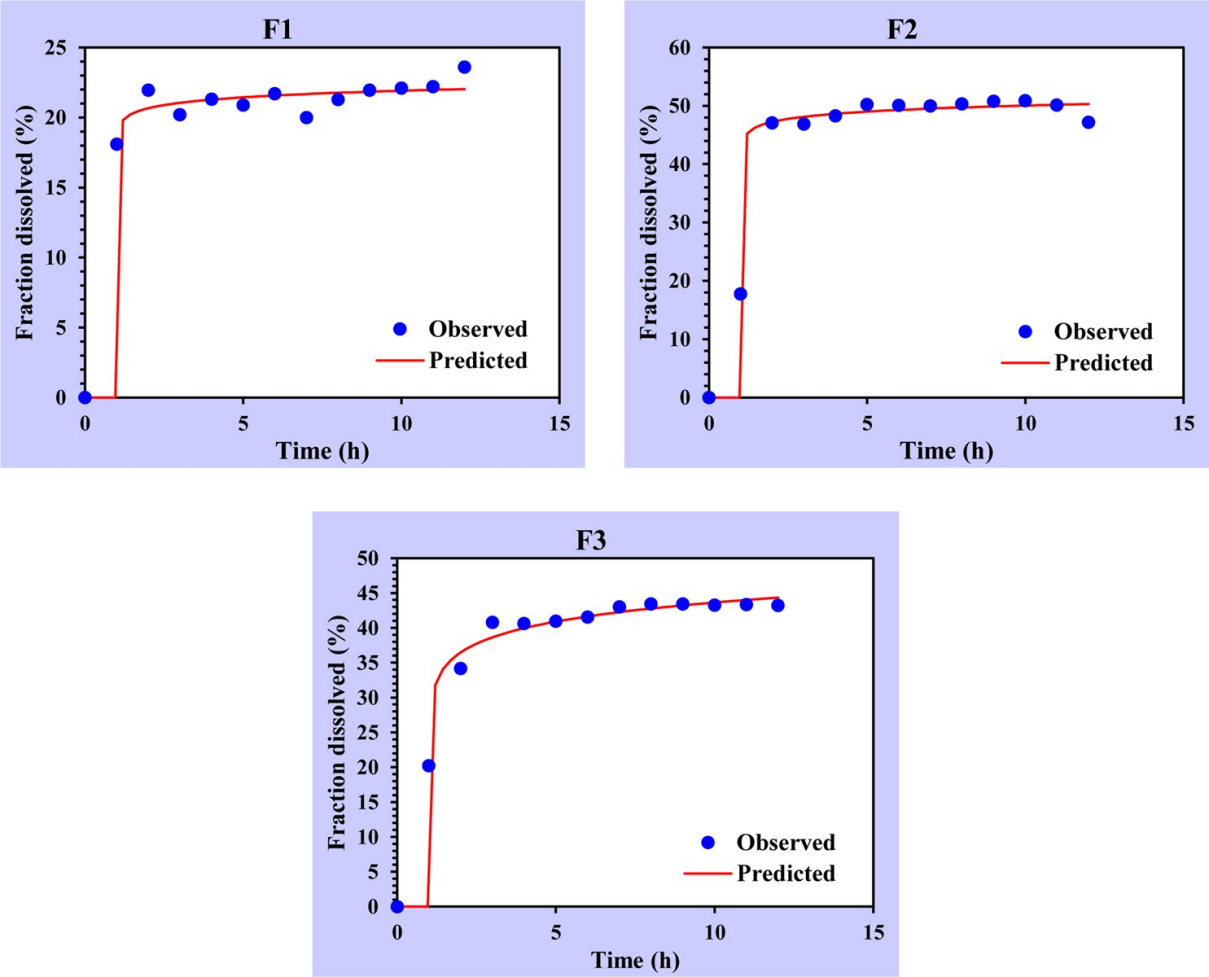
The correlation of the amount of PE released (Q_0_) versus the predicted amount of PE released (Qc) of F1, F2, and F3 by the Weibull model (Goodness of Fit).

Other goodness of fit (GOF) evaluations based on the correlation of Q_0_ vs. Q_c_ are backed by this analysis, as demonstrated in Fig 10.

The Weibull model exhibited the lowest aberration between the observed release data (Q_0_) and the predicted released data (Q_c_) and hence is confirmed to have the best fit for the phenolic compounds release data. Nevertheless, since the Weibull model is an empirical model that is not deduced from any kinetic fundament, it still presents some limitations [44].

Several criteria, including the previously analyzed AIC, MSC, and GoF, were considered in the selection of the candidate formula. According to the data, F2 had the highest MSC and lowest AIC values among the other formulas in the Weibull model, and the difference was statistically highly significant (*p*< 0.01). Moreover, it was visually clear that F2 had the least divergence between Q0 and Qc when compared to the other formulas (Fig 9), supporting the notion that it should be chosen as the optimum formula. However, to aid with this decision, the DDSolver was further used to generate simulated pharmacokinetic parameters such as AUC and MDT (Table 6).

It was found that the highest value of AUC was shown with F2, and the difference was statistically significant (*p*< 0.05), which is consistent with the results of EE% and *in vitro* release studies. Moreover, MDT is a tool for estimating the rate at which a drug is released from a dosage form lower values of MDT denote a more sustained release of the PE from the prepared sphingosomes. As noticed in Table 6, F2 exhibited an MDT of 0.642h, which is the shortest compared to F1 and F3 (*p*< 0.05). The finding of both of these parameters supports the selection of F2 as the candidate formula.

#### 3.6.6 Selection of the candidate formula

As mentioned earlier, many parameters were taken into consideration for the appropriate selection of the candidate formula. First of all, the *in vitro* release profile for the three formulas was studied. Although F1 showed a faster-sustained release profile, the amount of phenolic compounds released after 12 hours (Q12) was only 19.6±1.38%, and it had the lowest EE% compared to the other formulas, so F1 was eliminated. F2 and F3, on the contrary, had a good sustained release profile accompanied by much higher Q12 values of 42.5±9.44%, and 36.8±6.70%, respectively. In addition, F2 and F3 possessed greater EE% of 71.64±0.74% and 57.88±1.81%, respectively, and were therefore selected for further kinetics investigations by the DDSolver software.

A range of assessments was carried out to choose between F2 and F3 including, AIC, MSC, GOF, AUC, and MDT. Based on the outcome of these assessments, F2 was chosen as the best candidate formula, as discussed earlier. Subsequently, F2 was subjected to FTIR spectroscopy analysis to investigate any probable interaction between the PE and the lipids used in its formulation as well as electron microscopy for further shape and size characterization.

#### 3.6.7 FTIR spectroscopy analysis

**Fig 11:**
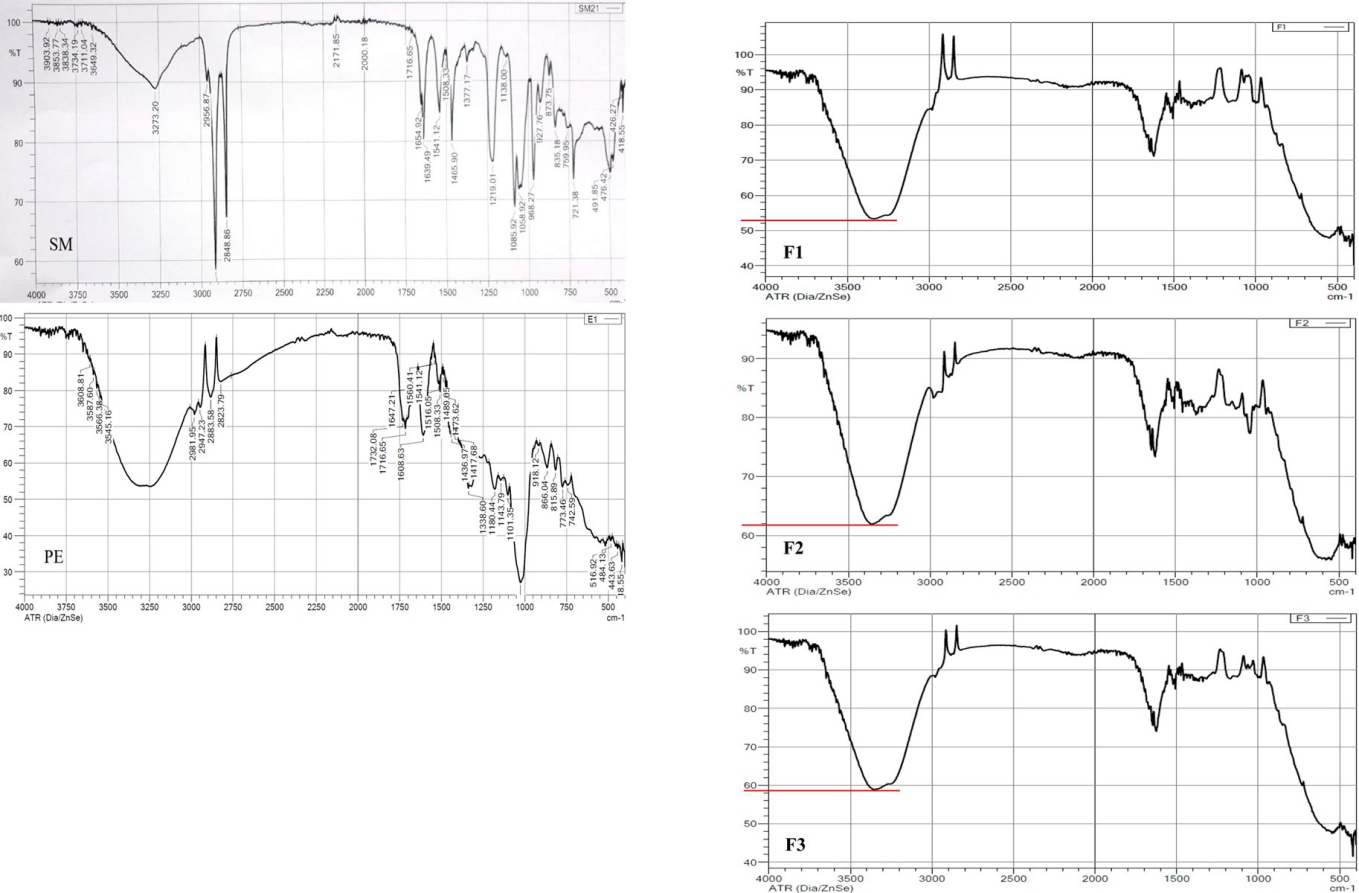
FTIR spectra of PE, SM, and F1-F3 sphingosomes.

The FTIR spectra of PE, pure SM, and the PE-loaded sphingosomal formulas (F1, F2, F3) are shown in Fig 11, and the absorption peaks are illustrated in Table 7.

**Table 7:** Characteristic peaks of FTIR spectra.

Functional group		Type of vibration	Peak frequency (cm^-1^)
**PE**	–OH	Stretch	3285.16
	C–H	Stretch	2883.58
	C = O	Stretch	1716.65
	C = C	Stretch	1608.63
	–CH_2_ or–CH_3_	Bend	1338.60
	C–O or C–N	Stretch	1026.13
**SM**	–OH	Stretch	3273.20
	C–H	Stretch	2916.37
		Stretch	2848.86
	CO–NH (amide)	Stretch	1654.92
		Stretch	1639.49
	Phosphodioxy group	Antisymmetric stretch	1219.01
		Symmetric stretch	1085.92
	C_5_H_14_NO (Choline)	-	968.27

When the spectra of PE and F1-F3 were compared, it was noted that the intensity of the–OH stretching peak of the PE spectrum (53%) was almost the same as that of the–OH peak in the F1 spectrum, but increased significantly in F2 and F3 spectra to 62% and 59%, respectively (*p*< 0.05). This increase in intensity may be attributed to the corresponding loading of PE into the vesicles, and since the F2 spectrum showed the greatest increase in the–OH peak intensity, loading was highest in F2, as evidenced by the EE % analysis. Moreover, the intensity of the C–H and C = C peaks of the PE spectrum was also increased in their corresponding peaks in F1, F2, and F3 spectra (from 78.17% to an average of 91.33% for C–H, and from 67.63% to an average of 72.67% for C = C peak). On the contrary, three peaks in the PE spectrum disappeared, namely C = O,–CH_2_ or–CH3, and C–O or C–N peaks.

On the other hand, when the spectra of SM and F1-F3 were compared, it was clear that five of the SM peaks disappeared in the spectra of the three formulas, particularly those belonging to the C–H, phosphodioxy, and choline groups. The–OH peak in SM was merged with that of the PE, and the intensity increased as explained earlier. The intensity of the CO–NH amide peaks between 1639.49cm^-1^ and 1654.92 cm^-1^, however, was reduced from 80.42–86.09% in the SM to 72.34%, 74.46%, and 75.55% in F1, F2, and F3 spectra, respectively.

Based on these findings, it was hypothesized that two mechanisms of PE entrapment existed. First, by incorporation of the PE into the vesicles, as indicated by the considerable increase in the intensity of the–OH peaks in the formulas’ spectra. Second, hydrogen bond interactions between the abundant nutraceuticals in PE and the lipids in the formulas, because all of these phytochemicals (phenolic acids, flavonoids, and anthocyanins) have aromatic rings and hydroxyl groups in their structures, which could be behind the disappearance of some of the PE and SM peaks.

#### 3.6.8 Scanning and Transmission Electron Microscopy (SEM and TEM)

**Fig 12:**
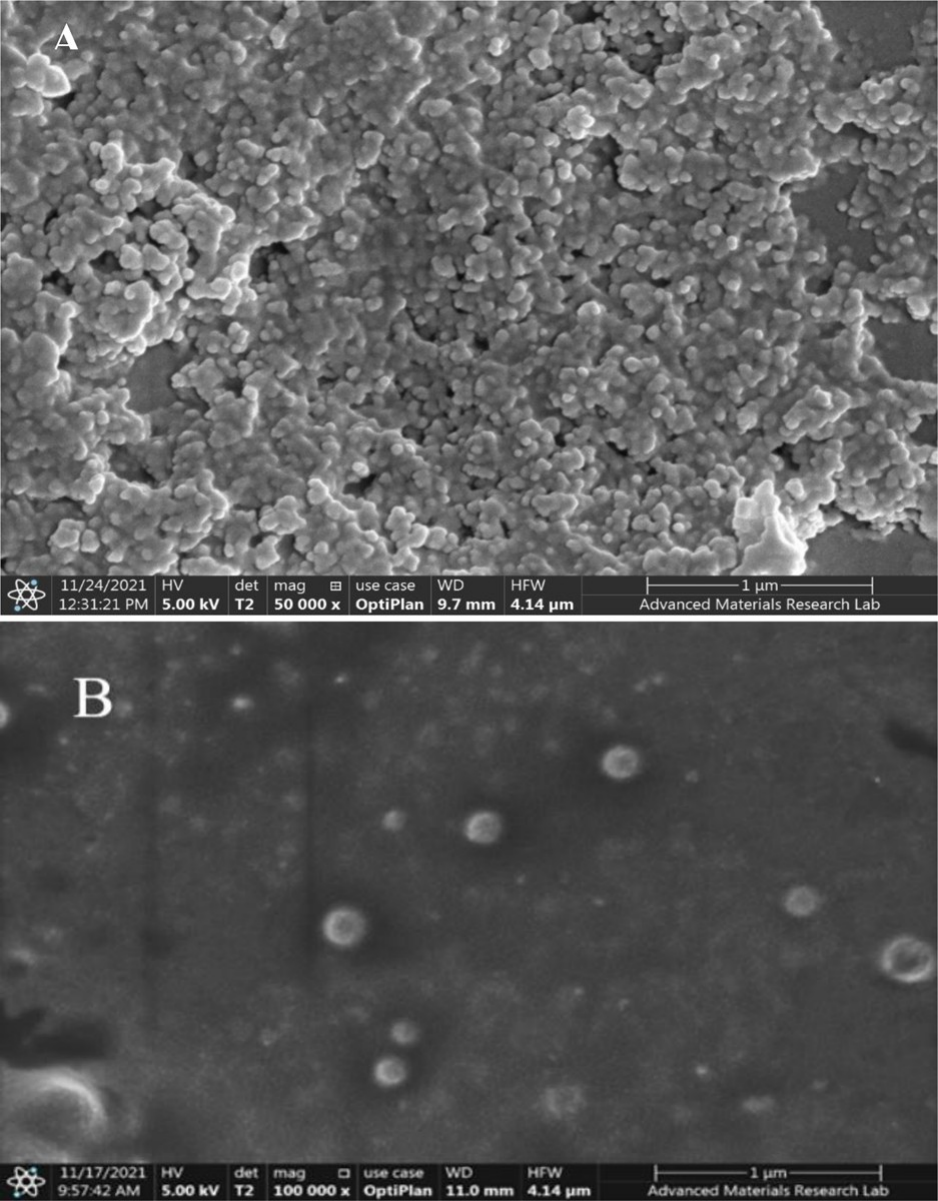
SEM micrographs of the prepared sphingosomes, A) Blank formula; B) Candidate formula (F2), at 50000X and 100000X, respectively.

To examine the morphology and surface topography of the selected F2, an SEM analysis was performed. The vesicles appeared to have a distinct and intact structure, with a smooth and homogenous surface, a spherical shape, and in the form of a non-porous lipid matrice, which supports the previously reported mechanism of phenolic compounds release by diffusion (Fig 12A and 12B).

Furthermore, for both the blank and candidate formulas, a TEM examination was carried out to establish the vesicle size, as this approach is thought to be the most effective in determining the actual size and internal structure of tested vesicles [45]. The GraphPad Prism 9.3.1.471 was used to analyze the data obtained, and the results were presented in Table 8.

**Table 8:** Statistical analysis results comparing the particle sizes of the blank and F2, obtained from TEM investigation (n = 3).

Parameter	Blank	F2
Mean	95.2033	*131.5
SD	3.0524	8.5106
SEM	1.7623	4.9136
n	3	3
*P*-value (two-tailed)	…….	0.0022

* Significant results at *p*< 0.05.

**Fig 13:**
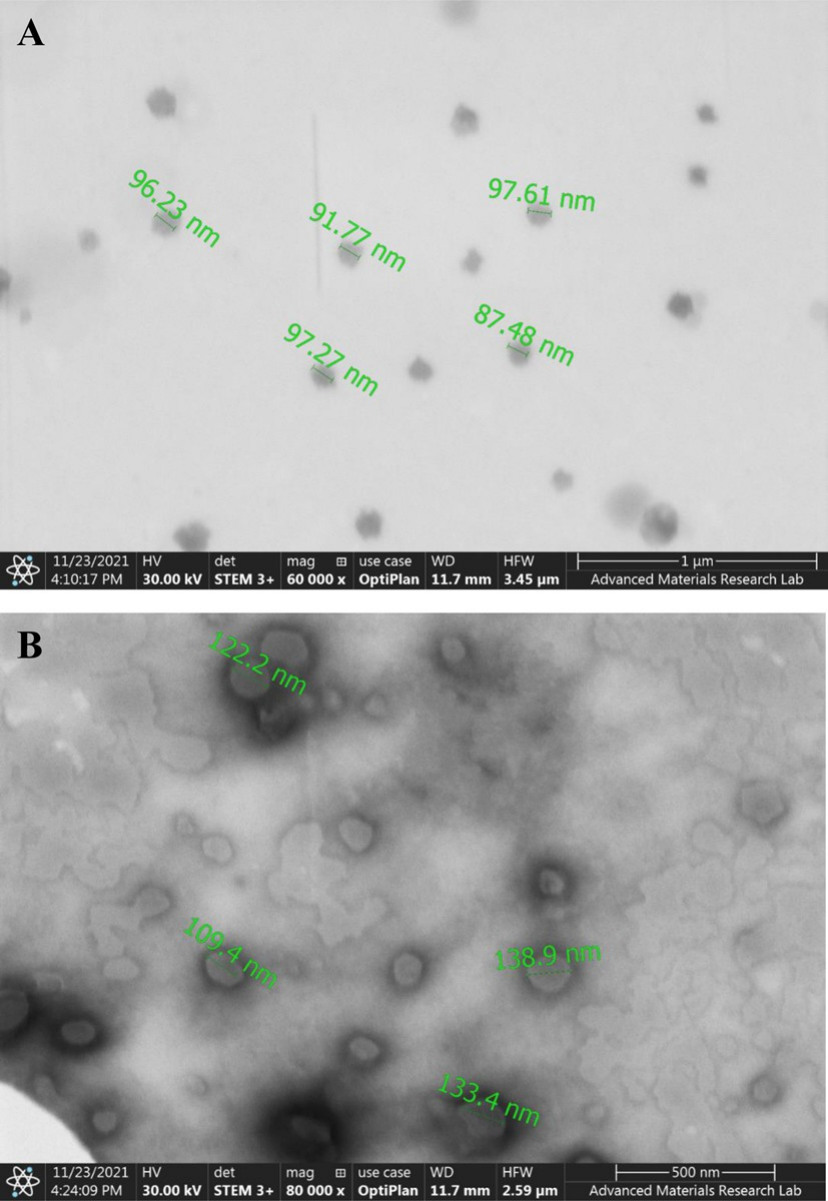
TEM micrographs of the prepared sphingosomes, A) Blank formula; B) Candidate formula (F2), at 60000X and 80000X, respectively.

It was observed that the PE-loaded sphingosomes of F2 had a significantly larger size than that of the blank (*p*< 0.01), confirming the PE loading into the vesicles (Fig 13A and 13B).

### 3.7 *In vitro* cytotoxicity study of PE and PE-loaded sphingosomes

In the current study, the anticancer activities of ethanolic and aqueous extracts of both pericarps and the juicy seeds of Spanish pomegranate fruits were compared to the control using MCF-7, HeLa, and HCT116 cancer cell lines. In addition, the anticancer activity of the candidate sphingosomal formulation -F2- was also tested using the same kinds of cell lines.

The MTT colorimetric assay was used to assess the cytotoxicity of the samples on the viability of the MCF-7, HeLa, and HCT116 cell lines. Under the conditions adopted for the study, the three cancer cell lines were treated with various concentrations (31.25, 62.5, 125, 250, 500, and 1000, μg/mL) of the four pomegranate extracts.

**Fig 14:**
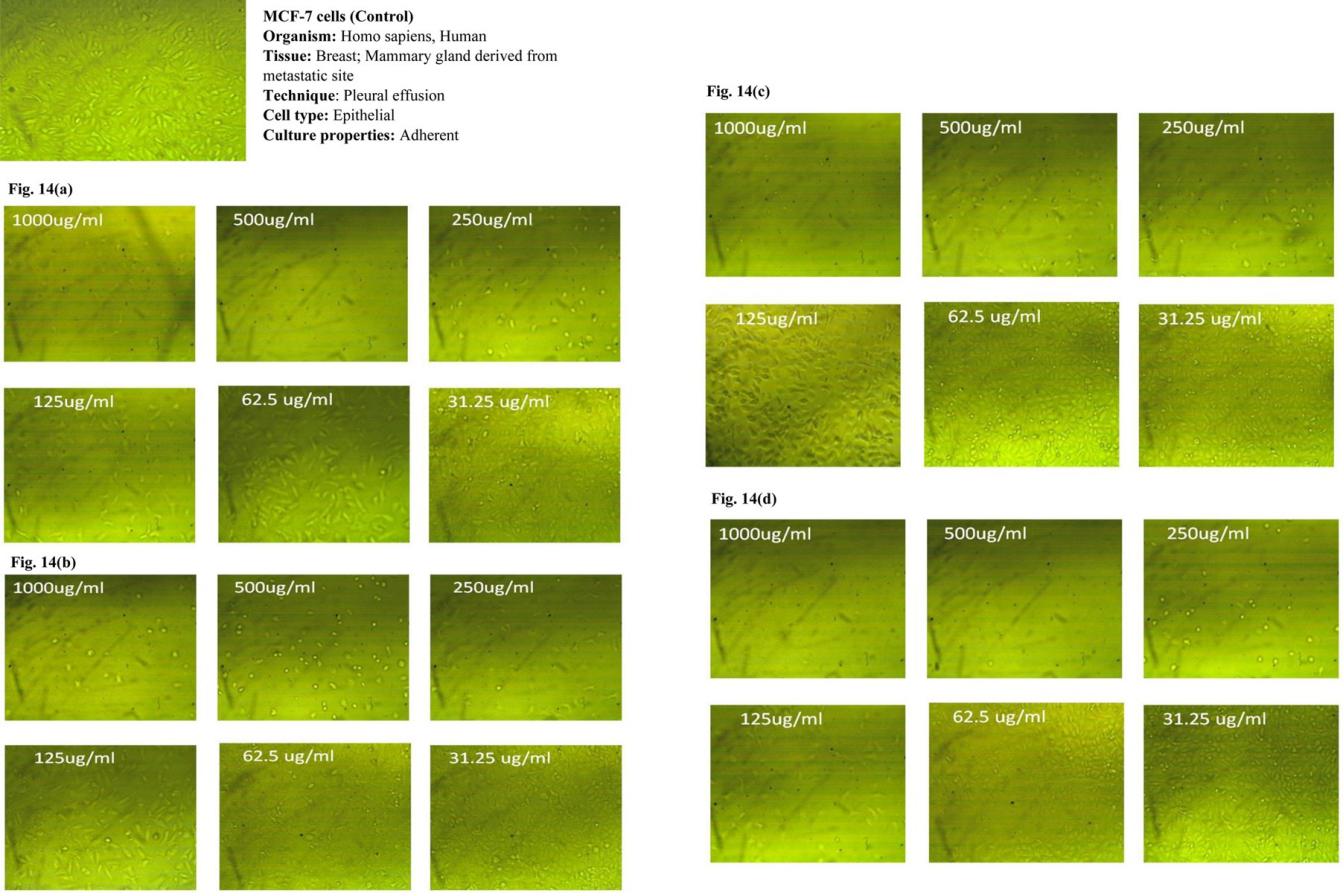
(a-d). Dose-dependent anticancer activity of plant extracts on MCF-7 cells at different concentrations compared to control cells. a) EPE; b) ASE; c) APE; d) ESE.

**Fig 15:**
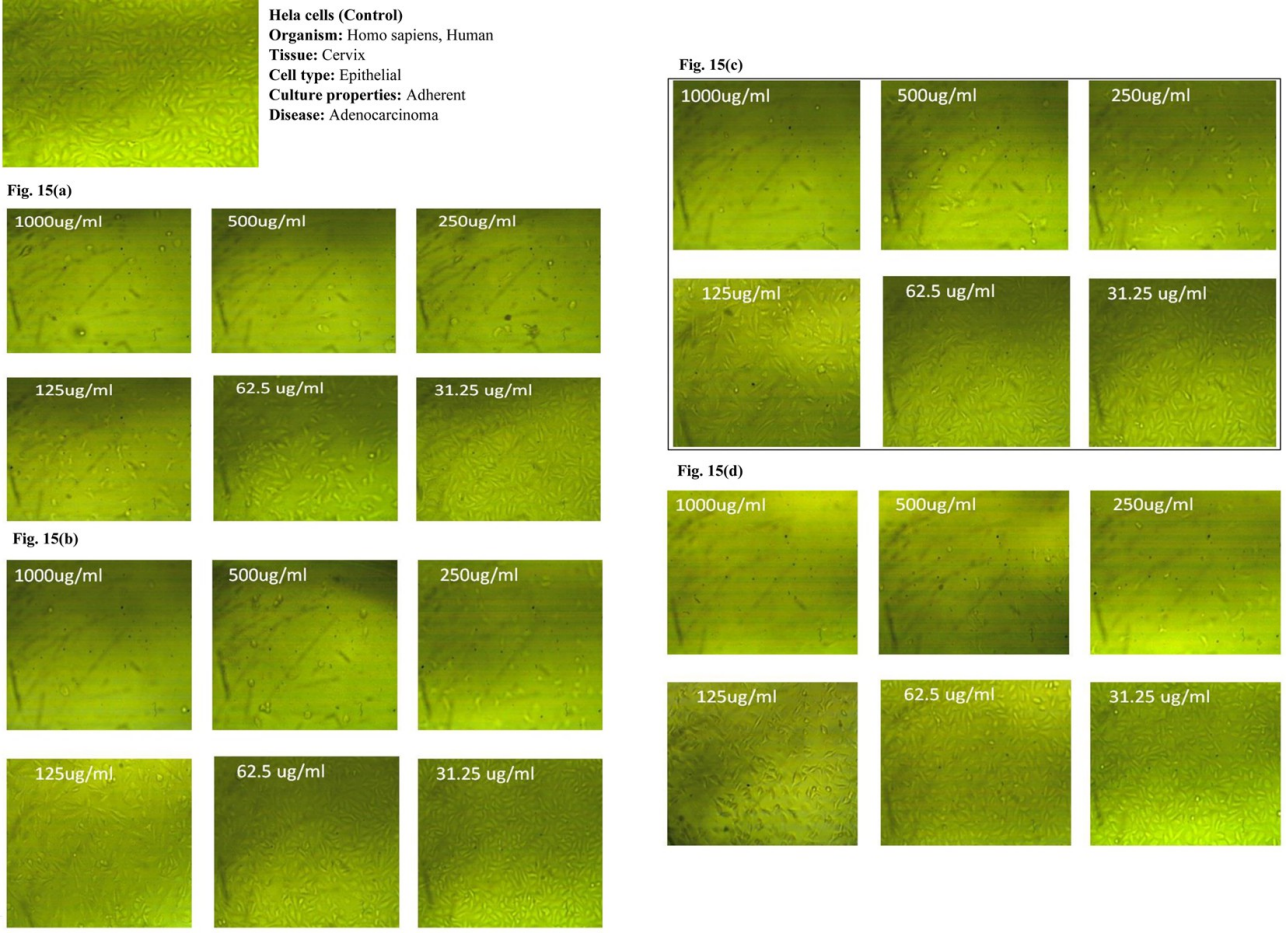
(a-d): Dose-dependent anticancer activity of plant extracts on Hela cells at different concentrations compared to control cells. a) EPE; b) ASE; c) APE; d) ESE.

**Fig 16:**
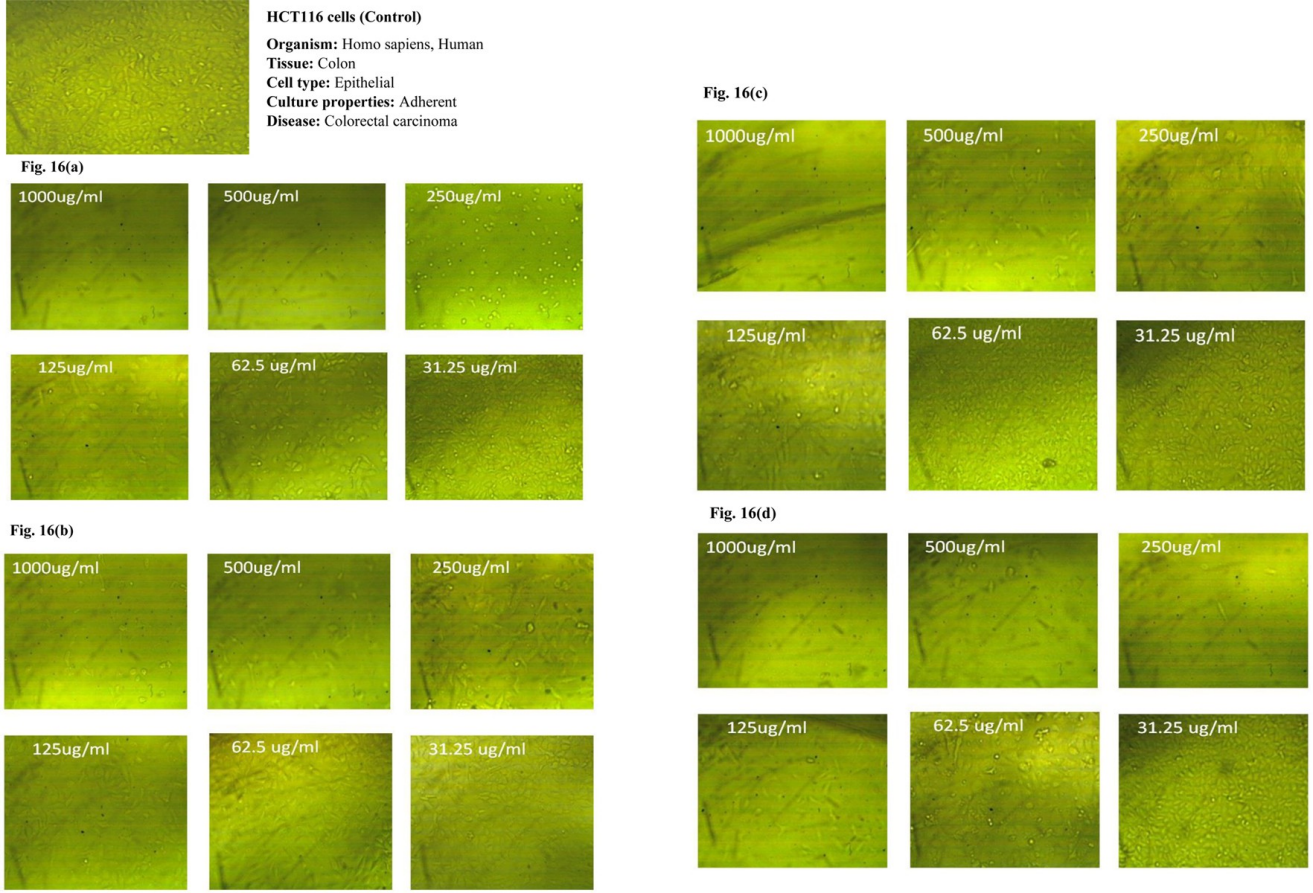
(a-d): Dose-dependent anticancer activity of plant extracts on HCT116 cells at different concentrations compared to control cells. a) EPE; b) ASE; c) APE; d) ESE.

The dose-dependent anticancer activity of plant extracts on MCF-7 HeLa, and HCT116 cell lines at different concentrations compared to control cells are displayed in Tables 9–11 and Figs 14(A)–14(D), 15(A)–15(D) and 16(A)–16(D), respectively.

**Table 9:** Cytotoxic effect and IC50 of the EPE, ASE, APE, and ESE on MCF-7 cell-line.

ID	Conc. μg/mL	Optical Density (O.D)			Mean O.D	SD	Viability %	Toxicity %	IC50 μg/mL ± SD
MCF7	Control	0.385	0.397	0.376	0.386	0.0061	100	0	
**EPE**	1000	0.019	0.020	0.020	0.020	0.0003	5.09	94.91	**58.22± 0.32**
	500	0.018	0.022	0.020	0.020	0.0012	5.18	94.82	
	250	0.035	0.062	0.044	0.047	0.0079	12.18	87.82	
	125	0.089	0.092	0.114	0.098	0.0079	25.47	74.53	
	62.5	0.163	0.158	0.172	0.164	0.0041	42.57	57.43	
	31.25	0.326	0.351	0.368	0.348	0.0122	90.24	9.76	
**ASE**	1000	0.035	0.04	0.036	0.037	0.0015	9.59	90.41	**121.47± 0.14**
	500	0.033	0.041	0.046	0.040	0.0038	10.36	89.64	
	250	0.05	0.081	0.079	0.070	0.0100	18.13	81.87	
	125	0.168	0.182	0.179	0.176	0.0043	45.68	54.32	
	62.5	0.362	0.345	0.358	0.355	0.0051	91.97	8.03	
	31.25	0.378	0.391	0.385	0.385	0.0038	99.65	0.35	
**APE**	1000	0.018	0.019	0.018	0.018	0.0003	4.75	95.25	**117.38± 0.69**
	500	0.053	0.042	0.038	0.044	0.0045	11.49	88.51	
	250	0.033	0.062	0.053	0.049	0.0086	12.78	87.22	
	125	0.163	0.158	0.172	0.164	0.0041	42.57	57.43	
	62.5	0.353	0.354	0.366	0.358	0.0042	92.66	7.34	
	31.25	0.385	0.389	0.379	0.384	0.0029	99.57	0.43	
**ESE**	1000	0.018	0.018	0.018	0.018	0.0000	4.66	95.34	**96.87± 0.51**
	500	0.02	0.019	0.019	0.019	0.0003	5.01	94.99	
	250	0.038	0.052	0.039	0.043	0.0045	11.14	88.86	
	125	0.114	0.092	0.126	0.111	0.0100	28.67	71.33	
	62.5	0.296	0.301	0.283	0.293	0.0054	75.99	24.01	
	31.25	0.397	0.372	0.388	0.386	0.0073	99.91	0.09	

EPE: Ethanolic pericarp extract; ASE: Aqueous seeds extract; APE: Aqueous pericarp extract; ESE: Ethanolic seeds extract, SD: standard deviation, IC50: half-maximal inhibitory concentration. SD: standard deviation, IC50: half-maximal inhibitory concentration.

**Table 10:** Cytotoxic effect and IC50 of the EPE, ASE, APE, and ESE on Hela cell-line.

ID	Conc. μg/mL	Optical Density (O.D)			Mean O.D	SD	Viability %	Toxicity %	IC50 μg/mL ± SD
Hela	Control	0.352	0.349	0.367	0.356	0.0056	100	0	
**EPE**	1000	0.020	0.019	0.019	0.019	0.0003	5.43	94.57	**56.62± 0.28**
	500	0.022	0.031	0.035	0.029	0.0038	8.24	91.76	
	250	0.045	0.036	0.030	0.037	0.0044	10.39	89.61	
	125	0.056	0.080	0.097	0.078	0.0119	21.82	78.18	
	62.5	0.153	0.142	0.123	0.139	0.0088	39.14	60.86	
	31.25	0.358	0.323	0.314	0.332	0.0134	93.16	6.84	
**ASE**	1000	0.019	0.02	0.02	0.020	0.0003	5.52	94.48	**162.94± 0.21**
	500	0.026	0.034	0.032	0.031	0.0024	8.61	91.39	
	250	0.056	0.071	0.088	0.072	0.0092	20.13	79.87	
	125	0.196	0.186	0.202	0.195	0.0047	54.68	45.32	
	62.5	0.324	0.345	0.347	0.339	0.0074	95.13	4.87	
	31.25	0.360	0.353	0.349	0.354	0.0032	99.44	0.56	
**APE**	1000	0.018	0.020	0.018	0.019	0.0007	5.24	94.76	**163.53± 0.32**
	500	0.045	0.038	0.051	0.045	0.0038	12.55	87.45	
	250	0.089	0.063	0.077	0.076	0.0075	21.44	78.56	
	125	0.196	0.152	0.187	0.178	0.0134	50.09	49.91	
	62.5	0.351	0.347	0.369	0.356	0.0068	99.91	0.09	
	31.25	0.345	0.355	0.368	0.356	0.0067	100.00	0.00	
**ESE**	1000	0.019	0.018	0.018	0.018	0.0003	5.15	94.85	**152.59± 0.45**
	500	0.020	0.026	0.025	0.024	0.0019	6.65	93.35	
	250	0.045	0.037	0.046	0.043	0.0028	11.99	88.01	
	125	0.156	0.182	0.187	0.175	0.0096	49.16	50.84	
	62.5	0.356	0.347	0.362	0.355	0.0044	99.72	0.28	
	31.25	0.359	0.362	0.347	0.356	0.0046	100.00	0.00	

EPE: Ethanolic pericarp extract; ASE: Aqueous seeds extract; APE: Aqueous pericarp extract; ESE: Ethanolic seeds extract, SD: standard deviation, IC50: half-maximal inhibitory concentration. SD: standard deviation, IC50: half-maximal inhibitory concentration.

**Table 11:** Cytotoxic effect and IC50 of the EPE, ASE, APE, and ESE on HCT116 cell-line.

ID	Conc. μg/mL	Optical Density (O.D)			Mean O.D	SD	Viability %	Toxicity %	IC50 μg/mL ± SD
HCT116	Control	0.384	0.399	0.375	0.386	0.007	100	0	
**EPE**	1000	0.019	0.02	0.018	0.019	0.0006	4.92	95.08	**94.79± 0.15**
	500	0.02	0.021	0.018	0.020	0.0009	5.09	94.91	
	250	0.056	0.063	0.058	0.059	0.0021	15.28	84.72	
	125	0.104	0.099	0.125	0.109	0.0080	28.32	71.68	
	62.5	0.265	0.284	0.279	0.276	0.0057	71.50	28.50	
	31.25	0.362	0.398	0.388	0.383	0.0107	99.14	0.86	
**ASE**	1000	0.018	0.017	0.019	0.018	0.0006	4.66	95.34	**177.6± 0.11**
	500	0.053	0.062	0.047	0.054	0.0044	13.99	86.01	
	250	0.115	0.121	0.108	0.115	0.0038	29.71	70.29	
	125	0.215	0.223	0.241	0.226	0.0077	58.64	41.36	
	62.5	0.341	0.362	0.358	0.354	0.0064	91.62	8.38	
	31.25	0.384	0.373	0.369	0.375	0.0045	97.24	2.76	
**APE**	1000	0.018	0.019	0.019	0.019	0.0003	4.84	95.16	**304.27± 0.32**
	500	0.035	0.056	0.057	0.049	0.0072	12.78	87.22	
	250	0.195	0.215	0.206	0.205	0.0058	53.20	46.80	
	125	0.348	0.369	0.375	0.364	0.0082	94.30	5.70	
	62.5	0.382	0.4	0.374	0.385	0.0077	99.83	0.17	
	31.25	0.393	0.378	0.385	0.385	0.0043	99.83	0.17	
**ESE**	1000	0.019	0.018	0.018	0.018	0.0003	4.75	95.25	**81.50± 0.84**
	500	0.02	0.019	0.021	0.020	0.0006	5.18	94.82	
	250	0.021	0.02	0.023	0.021	0.0009	5.53	94.47	
	125	0.056	0.063	0.068	0.062	0.0035	16.15	83.85	
	62.5	0.236	0.215	0.201	0.217	0.0102	56.30	43.70	
	31.25	0.385	0.397	0.371	0.384	0.0075	99.57	0.43	

EPE: Ethanolic pericarp extract; ASE: Aqueous seeds extract; APE: Aqueous pericarp extract; ESE: Ethanolic seeds extract, SD: standard deviation, IC50: half-maximal inhibitory concentration. SD: standard deviation, IC50: half-maximal inhibitory concentration.

It was observed that, for all tested cell lines, the cytotoxic effect of all pomegranate extracts varied in a dose-dependent pattern. The minimum antitumor effect was obtained at a dosage range of 31.25–62.5 μg/mL for all extracts, with a consistent increase in cancer cell inhibition as the concentration reached 1000 μg/mL, at which more than 90% growth inhibition was achieved in all tested cell lines. Furthermore, optical density, which correlates to the cancer cells’ quantity, was also recorded. An inverse relationship was noticed between the concentration of the extracts used and the value of optical density when compared to the untreated cells (control). In comparison to the other extracts, the ethanolic pericarps extract and the extract of the ethanolic seeds were shown to possess the strongest anticancer effects against MCT7, HeLa, and HCT116 cell lines at all concentrations tested. However, the former was superior against MCT and HeLa cell lines with the smallest IC_50_ (58.22 μg/mL and 56.62 μg/mL, respectively). While the latter displayed greater cytotoxic potency against the HCT116 cell line, with an IC_50_ value of 81.5 μg/mL. Our findings were in line with that of a study by Shalaby *et al*., where authors established that the pericarps and juice pomegranate extracts demonstrated the capability to inhibit tumor cell growth in multiple human cancer cell lines, including the MCF7 and HCT116 cell lines [46].

Moreover, the observed PEs’ anticancer effects against MCT7, HeLa, and HCT116 cell lines are most likely attributed to the presence of flavonoids such as kaempferol, rutin, hesperidin, and catechin, as well as phenolic compounds such as ellagic acid, pyrogallol, and ferulic acid, which, according to the literature, all have anticancer properties against the studied cell lines [47, 48]. The phenolic acids that are present in pomegranate extracts can be categorized into two groups: hydroxycinnamic acid (e.g. chlorogenic, caffeic, and ferulic acids) and hydroxybenzoic acid such as syringic and vanillic acids. Another important group of phytochemicals in pomegranates is the flavonoids which are now used in a wide range of medical, pharmaceutical, nutraceutical, and cosmetic products. There is evidence that these compounds can act as potent antioxidants, anticarcinogenic, antimutagenic, anti-diabetic, anti-ulcer, and anti-microbial agents [6].

Recently flavonoids have been reported to possess powerful inhibitory properties against a variety of enzymes, such as cyclooxygenase (COX), xanthine oxidase (XO), lipoxygenase (LOX), aldose reductase, and phosphoinositide 3-kinase [7]. Anthocyanins have been shown to have numerous health-promoting properties and may prevent some degenerative diseases. Also, anthocyanins can combat oxidative stress, function as antimicrobial agents, and prevent the emergence and progression of a wide range of noncommunicable diseases, including cardiovascular, metabolic, and neurodegenerative diseases, as well as certain types of cancer [9]. For instance, a range of pomegranate-derived substances was tested for their capacity to suppress aromatase activity, including gallagic acid, urolithins A and B, and ellagic acid. According to the findings, Urolithin B demonstrated the highest capacity, among others, to reduce aromatase activity that is responsible for stimulating the proliferation of estrogen-responsive breast tumors. Also, the pomegranate components were proved as antiangiogenesis agents through their marked suppression of the inflammatory or angiogenesis indicators (vascular endothelial growth factor (VEGF), in MDA-MB-231, MCF-7, and MCF-10A breast cancer cells [49].

Moreover, it has been demonstrated that the polyphenols in pomegranate fermented juices and seed oils can inhibit cancer cell growth and invasion. This effect was attributed to the inhibition of the oxidation and synthesis of proinflammatory prostaglandins that induce cell death in cancer [50]. Also, pomegranate polyphenols such as ellagitannins have a significant impact on colon cancer onset and progression [51]. It was observed that tumor necrosis factor-induced COX-2 protein synthesis was inhibited in HT-29 cancer cells treated with pomegranate juice. These findings highlighted the role of pomegranate juice in suppressing inflammatory signaling pathways in colonic malignancies [52]. In another study, pomegranate ellagic acid successfully induced Caco-2 colon cancer cellular apoptosis by activating the intrinsic apoptotic cascade. Surprisingly, however, this ellagic acid-induced apoptosis did not affect normal colonic cells [53]. Another research revealed the capacity of ellagic acid obtained from the extract of pomegranate peel to suppress the AKT/mTOR signaling cascade by altering IGFBP7 gene expression, with resultant inhibition of HeLa cancerous cells [54]. Other researchers suggested the potential cytotoxic, antiproliferation, and anti-invasion properties of the pomegranate peel extract against the tested HeLa cells. A dose-dependent anticancer impact of the aqueous pomegranate peel extract against the cervical cancer cell line was proposed [55].

Additionally, researchers found that pomegranate extract affects genes and proteins responsible for cancer growth and progression. In addition, pomegranate juices and pericarp have a three-fold higher antioxidant effect than green tea, and long before it was proven that the synergistic effect of polyphenols present in pomegranates explains the potent antioxidant and anticancer properties of the pericarp and seeds [56].

**Fig 17:**
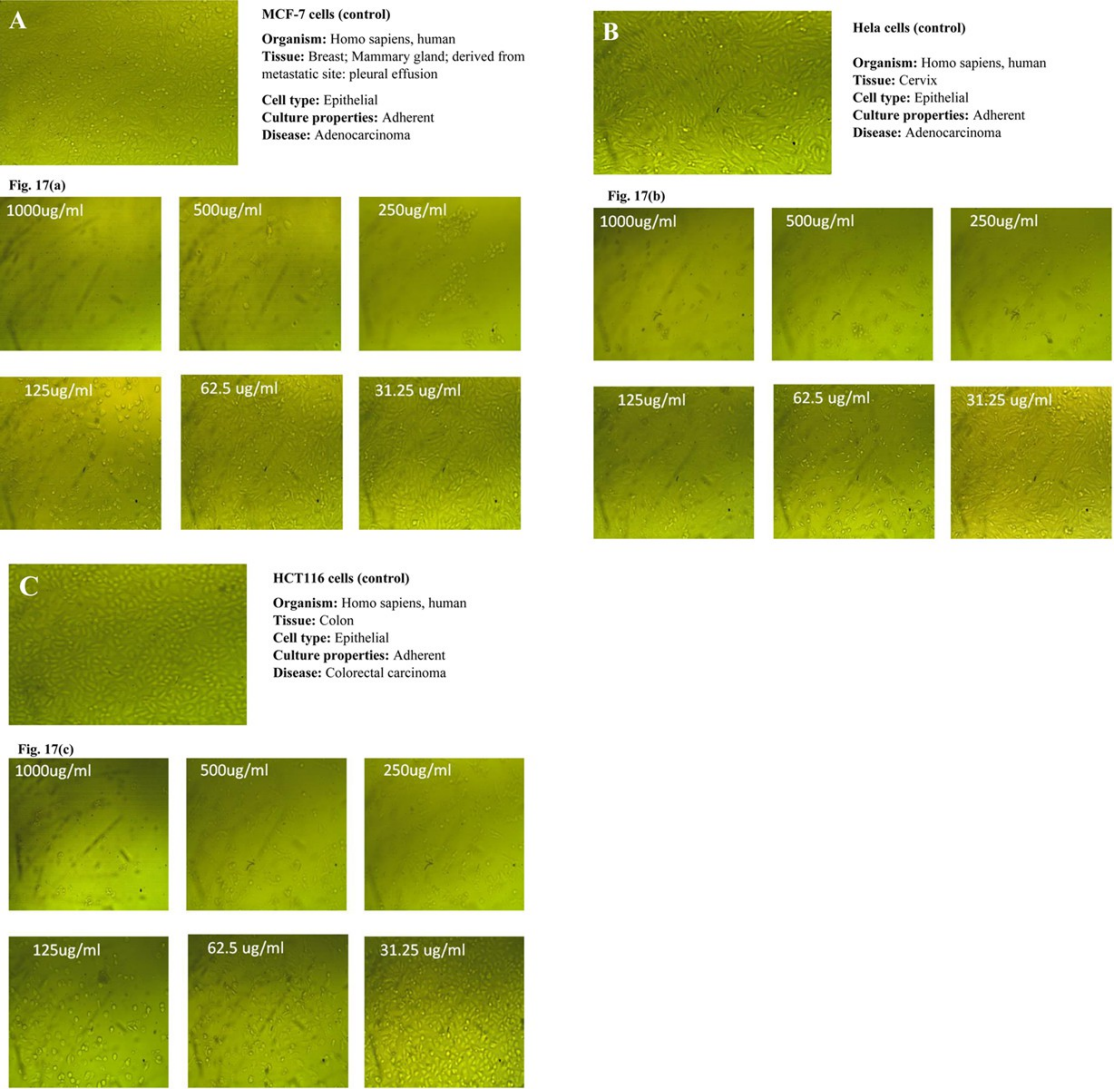
(a-c). Dose-dependent anticancer activity of the optimized formula F2 against a: MCF-7 cell lines, b: Hela cell lines, and c: HCT116 cell lines.

On the other hand, the MTT colorimetric assay was used to assess the cytotoxicity of the candidate formula on the viability of the MCF-7, HeLa, and HCT116 cell lines. Under the conditions adopted for the study, the three cancer cell lines were treated with various concentrations (31.25, 62.5, 125, 250, 500, and 1000, μg/mL) of F2, and Table 12 and Fig 17 (a)-(c) present the findings which explain why we had chosen F2 as a candidate promising formulation.

**Table 12:** Cytotoxic effect and IC50 of the candidate formula on MCF-7 cell line, Hela cell line, and HCT116 cell line.

ID	Conc. μg/mL	Optical Density (O.D)			Mean O.D	SD	Viability %	Toxicity %	IC50 μg/mL± SD
**MCF7**	**Control**	**0.866**	**0.872**	**0.878**	**0.872**	**0.0035**	**100**	**0**	**63.16± 0.64**
	1000	0.020	0.018	0.021	0.020	0.0009	2.26	97.74	
	500	0.045	0.052	0.058	0.052	0.0038	5.93	94.07	
	250	0.089	0.093	0.099	0.094	0.0029	10.74	89.26	
	125	0.213	0.235	0.221	0.223	0.0064	25.57	74.43	
	62.5	0.546	0.538	0.552	0.545	0.0041	62.54	37.46	
	31.25	0.821	0.856	0.843	0.840	0.0102	96.33	3.67	
**Hela**	**Control**	**0.754**	**0.768**	**0.77**	**0.764**	**0.0050**	**100**	**0**	**63.90± 0.72**
	1000	0.023	0.031	0.023	0.026	0.0027	3.36	96.64	
	500	0.022	0.031	0.028	0.027	0.0026	3.53	96.47	
	250	0.076	0.085	0.069	0.077	0.0046	10.03	89.97	
	125	0.214	0.22	0.247	0.227	0.0101	29.71	70.29	
	62.5	0.432	0.467	0.451	0.450	0.0101	58.90	41.10	
	31.25	0.768	0.737	0.725	0.743	0.0128	97.29	2.71	
**HCT116**	**Control**	**0.838**	**0.821**	**0.834**	**0.831**	**0.0051**	**100**	**0**	**54.79± 0.89**
	1000	0.017	0.018	0.019	0.018	0.0006	2.17	97.83	
	500	0.032	0.026	0.045	0.034	0.0056	4.13	95.87	
	250	0.068	0.085	0.077	0.077	0.0049	9.23	90.77	
	125	0.167	0.154	0.14	0.154	0.0078	18.49	81.51	
	62.5	0.376	0.398	0.381	0.385	0.0067	46.33	53.67	
	31.25	0.777	0.78	0.793	0.783	0.0049	94.26	5.74	

SD: standard deviation, IC50: half-maximal inhibitory concentration.

Similar to the outcome of the cytotoxicity study of the prepared extracts the anticancer effect of the candidate formula on all tested cell lines varied in a dose-dependent manner. The lowest antitumor impact was obtained at a dosage range of 31.25–62.5 μg/mL, with a consistent increase in cancer cell inhibition as the concentration reached 1000 μg/mL, at which more than 90% growth inhibition was achieved in all tested cell lines. Furthermore, optical density, which correlates to the cancer cells’ quantity, was also recorded. An inverse relationship was noticed between the concentration of the candidate formula used and the value of optical density when compared to the untreated cells (control).

In comparison to the anticytotoxic activity of the free extract (EPE), the candidate formula was shown to possess a stronger anticancer effect against HCT116 cell lines at all concentrations tested. This was evident through a significantly lower IC_50_ value of 54.79± 0.89 μg/mL compared to that of the free extract (IC_50_ of 94.79 μg/mL) (p<0.01). In addition, F2 exhibited statistically significant (p<0.05). cytotoxic impact against the MCF-7 and HeLa cell lines and HCT116 cell lines compared to the free extract. This may be attributed to the controlled release of the extract’s active substances from the prepared sphingosomes, which was confirmed in the *in vitro* study. This implies that the cancer cells were exposed to the extract for a longer time, hence achieving an enhanced cell inhibition effect.

Our findings were consistent with those of a study conducted by Saengkrit *et al*. where the anticancer effect of curcumin-loaded liposomes on HeLa and SiHa cervical cell lines was studied. The authors’ results demonstrated a more pronounced cytotoxic activity of the liposomal curcumin formulations in both cells, in comparison to the free curcumin [57]. Moreover, Arienti *et al*. developed liposomal cisplatin formulations (lipoplatin) and compared their anticancer activity as well as safety profile to those of pure cisplatin against cell lines derived from renal cell carcinoma, non-small cell lung cancer, and normal hematopoietic cell precursors [58]. Their findings established a superior cytotoxicity of Lipoplatin in all tumor cell types and significantly reduced toxicity in normal cells as compared to free cisplatin, supporting the use of these delivery vehicles in cancer therapy.

## 4. Conclusions

In the present study, four *Punica granatum* extracts were found to possess varying concentrations of flavonoids, phenolic compounds, and anthocyanins. They have all exhibited antioxidant and anticancer activities, too. However, the ethanolic pericarps extract demonstrated superior cytotoxic effects against MCT7 and HeLa cell lines and the second-best anticancer activity against HCT116 cell lines, with IC50 values of 58.22 μg/mL, 56.62 μg/mL, and 94.79 μg/mL, respectively. It also had the highest levels of flavonoids and phenolic acids (1.2% and 1.75%, respectively), as well as the second-highest levels of anthocyanins (99.97±0.17 mg/L) and the highest antioxidant activity (89.9%). As a result, the ethanolic pericarp extract was selected to proceed with the formulation production.

Following that, this PE was successfully loaded into sphingosomes consisting of SM and cholesterol. Different PE: lipids ratios were used to synthesize three sphingosomal formals by the thin-film hydration method. The preparation procedure was optimized by employing 8mL of chloroform and 2mL of methanol as solvents, 65° C and 100 rpm for solvent evaporation, 5mL of PBS as hydration medium, and two hrs as hydration time.

The selected candidate formula was F2, which was fabricated with 80.4mg of SM in a PE: lipids ratio of 1:2. It was chosen for its favorable *in vitro* sustained release profile, highest *in vitro* release (Q12) of 42.5±9.44%, and highest EE% of 71.64±0.74%. The F2 was also characterized by a suitable nanoparticle size of 131.5±4.91nm. Furthermore, the production of the sphingosomes in F2 was validated by SEM, and their successful loading with PE was confirmed by TEM size measurement. The FTIR analysis revealed that PE incorporation was the main mechanism of the drug loading into the lipid vesicles. On the other hand, using the DDSolver proved the best fit for the *in vitro* drug release data by the Weibull model. The shape parameter (β) was ≤0.75, indicating that the phenolic compounds were released from the sphingosomes following the Fickian diffusion mechanism. The simulated pharmacokinetic parameters investigated also portrayed F2 as the optimized formula. In addition, F2 exhibited a significant (p<0.05) stronger and prolonged anticancer effect against MCF-7, HeLa, and HCT116 cells at all concentrations tested compared to the free extract (EPE). Our research suggests that sphingosomes may prove to be an effective drug delivery system, improving pharmacological efficacy and reducing serious side effects of anticancer medications and natural products.

## Supporting information

S1 Graphical abstract (JPG)

## References

[1] Shaikh SB, Bhandary YP. Therapeutic properties of Punica granatum L (pomegranate) and its applications in lung-based diseases: A detailed review. J Food Biochem. 2021 Apr;45(4):e13684. doi: 10.1111/jfbc.13684 33709449

[2] Caruso A, Barbarossa A, Tassone A, Ceramella J, Carocci A, Catalano A, et al. Pomegranate: nutraceutical with promising benefits on human health. Appl. Sci. 2020; 10:6915. doi: 10.3390/app10196915

[3] Kandylis P, Kokkinomagoulos E. Food Applications and Potential Health Benefits of Pomegranate and its Derivatives. Foods. 2020 Jan 23;9(2):122. doi: 10.3390/foods9020122 31979390 PMC7074153

[4] Mattioli R, Francioso A, Mosca L, Silva P. Anthocyanins: A Comprehensive Review of Their Chemical Properties and Health Effects on Cardiovascular and Neurodegenerative Diseases. Molecules. 2020 Aug 21;25(17):3809. doi: 10.3390/molecules25173809 32825684 PMC7504512

[5] Bassiri-Jahromi S. Punica granatum (Pomegranate) activity in health promotion and cancer prevention. Oncol Rev. 2018 Jan 30;12(1):345. doi: 10.4081/oncol.2018.345 29441150 PMC5806496

[6] Hong MY, Seeram NP, Heber D. Pomegranate polyphenols down-regulate expression of androgen-synthesizing genes in human prostate cancer cells overexpressing the androgen receptor. J Nutr Biochem. 2008 Dec;19(12):848–55. doi: 10.1016/j.jnutbio.2007.11.006 18479901 PMC2610864

[7] Pandey M, Choudhury H, Gorain B, Tiong SQ, Wong GYS, Chan KX, et al. Site-Specific Vesicular Drug Delivery System for Skin Cancer: A Novel Approach for Targeting. Gels. 2021 Nov 16;7(4):218. doi: 10.3390/gels7040218 34842689 PMC8628733

[8] Saraf S, Paliwal S, Kaur CD, Saraf SK. Sphingosomes a novel approach to vesicular drug delivery. Research Journal of Pharmacy and Technology. 2011;4:661–666.

[9] Chaudhari SP, Gaikwad SU. Sphingosomes: A novel lipoidal vesicular drug delivery system. J Sci Technol. 2020 Apr 6;5(4):261–7. doi: 10.46243/jst.2020.v5.i4.pp261-267

[10] Liu P, Chen G, Zhang J. A Review of Liposomes as a Drug Delivery System: Current Status of Approved Products, Regulatory Environments, and Future Perspectives. Molecules. 2022 Feb 17;27(4):1372. doi: 10.3390/molecules27041372 35209162 PMC8879473

[11] Lopez C, David-Briand E, Mériadec C, Bourgaux C, Pérez J, Artzner F. Milk sphingosomes as lipid carriers for tocopherols in aqueous foods: Thermotropic phase behaviour and morphology. Food Res Int. 2022 Dec;162(Pt B):112115. doi: 10.1016/j.foodres.2022.112115 36461349

[12] Lopez C, Mériadec C, David-Briand E, Dupont A, Bizien T, Artzner F, et al. Loading of lutein in egg-sphingomyelin vesicles as lipid carriers: Thermotropic phase behaviour, structure of sphingosome membranes and lutein crystals. Food Res Int. 2020 Dec;138(Pt A):109770. doi: 10.1016/j.foodres.2020.109770 33292950

[13] Bedikian AY, Vardeleon A, Smith T, Campbell S, Namdari R. Pharmacokinetics and urinary excretion of vincristine sulfate liposomes injection in metastatic melanoma patients. J Clin Pharmacol. 2006 Jul;46(7):727–37. doi: 10.1177/0091270006288953 16809798

[14] Singleton VL, Rossi JA. Colorimetry of total phenolics with phosphomolybdic-phosphotungstic acid reagents. Am J Enol Vitic. 1965;16(3):144–158.

[15] Unir OM, Ufrevio IG, Glu IK. Determination of in vitro antioxidant activity of fennel (*Foeniculum vulgare*) seed extracts. LWT—Food Sci Technol. 2003;36:263–71. doi: 10.1016/S0023-6438(02)00226-8

[16] Dewanto V, Wu X, Adom KK, Rui A, Liu H. Thermal processing enhances the nutritional value of tomatoes by increasing total antioxidant activity. J Agric Food Chem. 2002;50(10):3010–3014. doi: 10.1021/jf0115589 11982434

[17] Román T, Larcher R, Slaghenaufi D, Tonidandel L, Moser S, Nicolini G. Anthocyanin profile and antioxidant activity of freshly squeezed pomegranate (Punica granatum L.) juices of Sicilian and Spanish provenances. Ital J Food Sci. 2016;28(3):464–79. doi: 10.14674/1120-1770/ijfs.v332

[18] Shehab N.G.; Abu-Gharbieh E.; Bayoumi F.A. Impact of phenolic composition on hepatoprotective and antioxidant effects of four desert medicinal plants. BMC Complement Altern Med. 2015. 9,;15:401. doi: 10.1186/s12906-015-0919-6 26552870 PMC4640355

[19] Lin Y. L.; Juan I. M.; Chen Y.L.; Liang Y.C.; Lin J.K. Composition of polyphenols in fresh tea leaves and associations of their oxygen-radical-absorbing capacity with antiproliferative actions in fibroblast cells. J Agric Food Chem (USA). 1997;44(6):1387–1394. doi: 10.1021/jf950652k

[20] Kuntić V.; Pejić N; Ivković B.; Vujić Z; Ilić K.; Mićić S.;et al. Isocratic RP-HPLC method for rutin determination in solid oral dosage forms. J Pharm Biomed Anal. 2007;43(2):718–21. doi: 10.1016/j.jpba.2006.07.019 16920326

[21] Van de Loosdrecht A.A.; Beelen R.H.J.; Ossenkoppele G.J.; Broekhoven M.G.; Langenhuijsen M.M. A tetrazolium-based colorimetric MTT assay to quantitate human monocyte-mediated cytotoxicity against leukemic cells from cell lines and patients with acute myeloid leukemia. J Immunol Methods. 1994, 14, 174(1–2), 311–20. doi: 10.1016/0022-1759(94)90034-5 8083535

[22] Alley M.C, Scudiero DA, Monks A, Hursey ML, Czerwinski MJ, Fine DL, et al. Feasibility of drug screening with panels of human tumor cell lines using a microculture tetrazolium assay. Cancer Res. 1988;48(3):589–601. 3335022

[23] Khalifa AM, Abdul Rasool BK. Optimized Mucoadhesive Coated Niosomes as a Sustained Oral Delivery System of Famotidine. AAPS PharmSciTech. 2017 Nov;18(8):3064–3075. doi: 10.1208/s12249-017-0780-7 28516414

[24] Walunj M, Doppalapudi S, Bulbake U, Khan W. Preparation, characterization, and *in vivo* evaluation of cyclosporine cationic liposomes for the treatment of psoriasis. J Liposome Res. 2020 Mar;30(1):68–79. doi: 10.1080/08982104.2019.1593449 30897993

[25] Abdelwahd A, Abdul Rasool BK. Optimizing and Evaluating the Transdermal Permeation of Hydrocortisone Transfersomes Formulation Based on Digital Analysis of the In Vitro Drug Release and Ex Vivo Studies. Recent Adv Drug Deliv Formul. 2022;16(2):122–144. doi: 10.2174/2667387816666220608115605 35676851 PMC10186384

[26] Barchan A, Bakkali M, Arakrak A, Pagán R, Laglaoui A.The effects of solvents polarity on the phenolic contents and antioxidant activity of three Mentha species extracts. IJCMAS. 2014;3(11):399–412. ISSN:2319-7692.

[27] Ali SI, El-Baz FK, El-Emary GA, Khan EA, Amal AM, Mohamed. HPLC-analysis of polyphenolic compounds and free radical scavenging activity of pomegranate fruit (Punica granatum L.). 2014. Available on: https://www.semanticscholar.org/paper/HPLC-Analysis-of-Polyphenolic-Compounds-and-Free-of-Ali-El-Baz/fe9b748351d2b5f1353b946e3355357e0ac2736f

[28] Derakhshan Z, Ferrante M, Tadi M, Ansari F, Heydari A, Sadat HM, et al. Antioxidant activity and total phenolic content of ethanolic extract of pomegranate peels, juice and seeds. Food and Chemical Toxicology. 2018;114: 108–111. doi: 10.1016/j.fct.2018.02.023 29448088

[29] Toledo-Machado CM, Bueno LL, Menezes-Souza D. Use of Phage Display technology in development of canine visceral leishmaniasis vaccine using synthetic peptide trapped in sphingomyelin/cholesterol liposomes. Parasites Vectors. 2015;8:133. doi: 10.1186/s13071-015-0747-z 25889286 PMC4352561

[30] Ong SG, Chitneni M, Lee KS, Ming LC, Yuen KH. Evaluation of Extrusion Technique for Nanosizing Liposomes. Pharmaceutics. 2016 Dec 21;8(4):36. doi: 10.3390/pharmaceutics8040036 28009829 PMC5198018

[31] Shaker S, Gardouh AR, Ghorab MM. Factors affecting liposomes particle size prepared by ethanol injection method. Res Pharm Sci. 2017 Oct;12(5):346–352. doi: 10.4103/1735-5362.213979 28974972 PMC5615864

[32] Danaei M, Dehghankhold M, Ataei S, Hasanzadeh Davarani F, Javanmard R, Dokhani A, et al. Impact of Particle Size and Polydispersity Index on the Clinical Applications of Lipidic Nanocarrier Systems. Pharmaceutics. 2018 May 18;10(2):57. doi: 10.3390/pharmaceutics10020057 29783687 PMC6027495

[33] Joseph E and Singhvi G. (2019). Multifunctional nanocrystals for cancer therapy: a potential nanocarrier. Nanomaterials for Drug Delivery and Therapy, pp. 91–116. doi: 10.1016/B978-0-12-816505-8.00007-2

[34] Lu GW and Gao P. (2010). Emulsions and Microemulsions for Topical and Transdermal Drug Delivery. Handbook of Non-Invasive Drug Delivery Systems, pp. 59–94. doi: 10.1016/B978-0-8155-2025-2.10003-4

[35] Fanani ML, Maggio B. The many faces (and phases) of ceramide and sphingomyelin I—single lipids. Biophys Rev. 2017 Oct;9(5):589–600. doi: 10.1007/s12551-017-0297-z 28815463 PMC5662039

[36] Calvagno MG, Celia C, Paolino D, Cosco D, Iannone M, Castelli F, et al. Effects of lipid composition and preparation conditions on physical-chemical properties, technological parameters and in vitro biological activity of gemcitabine-loaded liposomes. Curr Drug Deliv. 2007 Jan;4(1):89–101. doi: 10.2174/156720107779314749 17269921

[37] Mao W, Wu F, Lee RJ, Lu W, Wang J. Development of a stable single-vial liposomal formulation for vincristine. Int J Nanomedicine. 2019 Jun 21;14:4461–4474. doi: 10.2147/IJN.S205276 31296986 PMC6596348

[38] Bazak R, Houri M, Achy SE, Hussein W, Refaat T. Passive targeting of nanoparticles to cancer: A comprehensive review of the literature. Mol Clin Oncol. 2014 Nov;2(6):904–908. doi: 10.3892/mco.2014.356 25279172 PMC4179822

[39] Semple SC, Leone R, Wang J, Leng EC, Klimuk SK, Eisenhardt ML, et al. Optimization and characterization of a sphingomyelin/cholesterol liposome formulation of vinorelbine with promising antitumor activity. J Pharm Sci. 2005 May;94(5):1024–38. doi: 10.1002/jps.20332 15793796

[40] Thomas DA, Sarris AH, Cortes J, Faderl S, O’Brien S, Giles FJ, et al. Phase II study of sphingosomal vincristine in patients with recurrent or refractory adult acute lymphocytic leukemia. Cancer. 2006 Jan 1;106(1):120–7. Erratum in: Cancer. doi: 10.1002/cncr.21595 16331634

[41] Justyna Kobryń Sandra Sowa, Gasztych Monika, Andrzej Dryś Witold Musiał, "Influence of hydrophilic polymers on the factor in weibull equation applied to the release kinetics of a biologically active complex of aesculus hippocastanum. Int. J. Polym. Sci. 2017, Article ID 3486384, 8 pages, 2017. doi: 10.1155/2017/3486384

[42] Abdul Rasool BK, Sammour R. DDSolver Software Application for Quantitative Analysis of In vitro Drug Release Behavior of the Gastroretentive Floating Tablets Combined with Radiological Study in Rabbits. Curr Drug Deliv. 2022 Aug 6;19(9):949–965. doi: 10.2174/1567201819666220304203014 35249487

[43] Shi Y, Wan A, Shi Y, Zhang Y, Chen Y. Experimental and mathematical studies on the drug release properties of aspirin loaded chitosan nanoparticles. Biomed Res Int. 2014;2014:613619. doi: 10.1155/2014/613619 24987696 PMC4058851

[44] Kalam MA, Amin S, Sultana Y. Release kinetics of modified pharmaceutical dosage forms: A review squamous cell carcinoma of skin view project formulation design view project. 2007, Available at: https://www.researchgate.net/publication/222711448 (accessed 17 January 2022).

[45] Orasugh JT, Ghosh SK, Chattopadhyay D. Nanofiber-reinforced biocomposites, Fiber-Reinforced Nanocomposites: Fundamentals and Applications, 2020, pp. 199–233. doi: 10.1016/B978-0-12-819904-6.00010-4

[46] Shalaby MT, Dawood DH, Hefnil DM, Murad BM. Phytochemical constituents, antimicrobial and antitumor effects of pomegranate fruit (*Punica granatum L*). JFDS. 2019;10(10):373–380. doi: 10.21608/jfds.2019.60208

[47] Kopustinskiene DM, Jakstas V, Savickas A, Bernatoniene J. Flavonoids as Anticancer Agents. Nutrients. 2020 Feb 12;12(2):457. doi: 10.3390/nu12020457 32059369 PMC7071196

[48] Rodríguez L, Trostchansky A, Vogel H, Wood I, Palomo I, Wehinger S, et al. A comprehensive literature review on cardioprotective effects of bioactive compounds present in fruits of Aristotelia Chilensis Stuntz (Maqui). Molecules. 2022 Sep 20;27(19):6147. doi: 10.3390/molecules27196147 36234679 PMC9571323

[49] Turrini E, Ferruzzi L, Fimognari C. Potential Effects of Pomegranate Polyphenols in Cancer Prevention and Therapy. Oxid Med Cell Longev. 2015;2015:938475. doi: 10.1155/2015/938475 26180600 PMC4477247

[50] Adams L.S; Zhang Y.; Seeram N.P.; Heber D.; Chen S. Pomegranate ellagitannin-derived compounds exhibit antiproliferative and antiaromatase activity in breast cancer cells in vitro. Cancer Prev Res (Phila). 2010, 3(1), 108–13. doi: 10.1158/1940-6207.CAPR-08-0225 20051378 PMC2805471

[51] Toi M.; Bando H.; Ramachandran C.; Melnick S.J.; Imai A.; Fife R.S; et al. Preliminary studies on the anti-angiogenic potential of pomegranate fractions in vitro and in vivo. Angiogenesis. 2003, 6(2),121–8. doi: 10.1023/B:AGEN.0000011802.81320.e4:AGEN.0000011802.81320.e4 14739618

[52] Kim; Mehta R.; Yu W., Neeman I.; Livney T.; Amichay A.; et al. Chemopreventive and adjuvant therapeutic potential of pomegranate (Punica granatum) for human breast cancer. Breast Cancer Res Treat. 2002, 71(3), 203–17. doi: 10.1023/a:1014405730585:1014405730585 12002340

[53] Syed D.N.; Chamcheu J.C.; Adhami V.M.; Mukhtar H. Pomegranate extracts and cancer prevention: molecular and cellular activities. Anticancer Agents Med Chem. 2013,13(8),1149–61. doi: 10.2174/1871520611313080003 23094914 PMC4052369

[54] Sharma P.; McClees S.F.; Afaq F. Pomegranate for prevention and treatment of cancer: An update. Molecules. 2017, 24, 22(1), 177. doi: 10.3390/molecules22010177 28125044 PMC5560105

[55] Larrosa M.; Tomás-Barberán F.A; Espín J.C. The dietary hydrolysable tannin punicalagin releases ellagic acid that induces apoptosis in human colon adenocarcinoma Caco-2 cells by using the mitochondrial pathway. J Nutr Biochem. 2006, 17(9), 611–25. doi: 10.1016/j.jnutbio.2005.09.004 16426830

[56] Guo H.; Zhang D.; Fu Q. Inhibition of cervical cancer by promoting IGFBP7 expression using ellagic acid from pomegranate peel. Med Sci Monit. 2016, 12, 22:4881–4886. doi: 10.12659/msm.898658 27941714 PMC5158133

[57] Saengkrit N, Saesoo S, Srinuanchai W, Phunpee S, Ruktanonchai UR. Influence of curcumin-loaded cationic liposome on anticancer activity for cervical cancer therapy. Colloids Surf B Biointerfaces. 2014 Feb 1;114:349–56. doi: 10.1016/j.colsurfb.2013.10.005 24246195

[58] Arienti C, Zoli W, Pignatta S, Carloni S, Paganelli G, Ulivi P, et al. Efficacy of different sequences of radio- and chemotherapy in experimental models of human melanoma. J Cell Physiol. 2014 Oct;229(10):1548–56. doi: 10.1002/jcp.24598 24591063

