# Correction: Pomegranate extract-loaded sphingosomes for the treatment of cancer: Phytochemical investigations, formulation, and antitumor activity evaluation

**DOI:** 10.1371/journal.pone.0307960

**Published:** 2024-07-24

**Authors:** Huda Jamal AlMadalli, Bazigha K. Abdul Rasool, Naglaa Gamil Shehab, Francesca Della Sala, Assunta Borzacchiello

There is an error in the article XML causing the fourth author’s name to be indexed incorrectly. The name should be indexed as Della Sala F. The correct citation is: AlMadalli HJ, Abdul Rasool BK, Shehab NG, Della Sala F, Borzacchiello A (2024) Pomegranate extract-loaded sphingosomes for the treatment of cancer: Phytochemical investigations, formulation, and antitumor activity evaluation. PLoS ONE 19(2): e0293115. https://doi.org/10.1371/journal.pone.0293115.

## References

[1] AlMadalli HJ, Abdul Rasool BK, Shehab NG, Sala FD, Borzacchiello A (2024) Pomegranate extract-loaded sphingosomes for the treatment of cancer: Phytochemical investigations, formulation, and antitumor activity evaluation. PLoS ONE 19(2): e0293115. doi: 10.1371/journal.pone.0293115 38346085 PMC10861072

