# Supplementary figures and images for: Pomegranate extract-loaded sphingosomes for the treatment of cancer: Phytochemical investigations, formulation, and antitumor activity evaluation
Source: PLoS One. 2024 Feb 12;19(2):e0293115. doi: 10.1371/journal.pone.0293115 (PMC10861072; doi:10.1371/journal.pone.0293115)

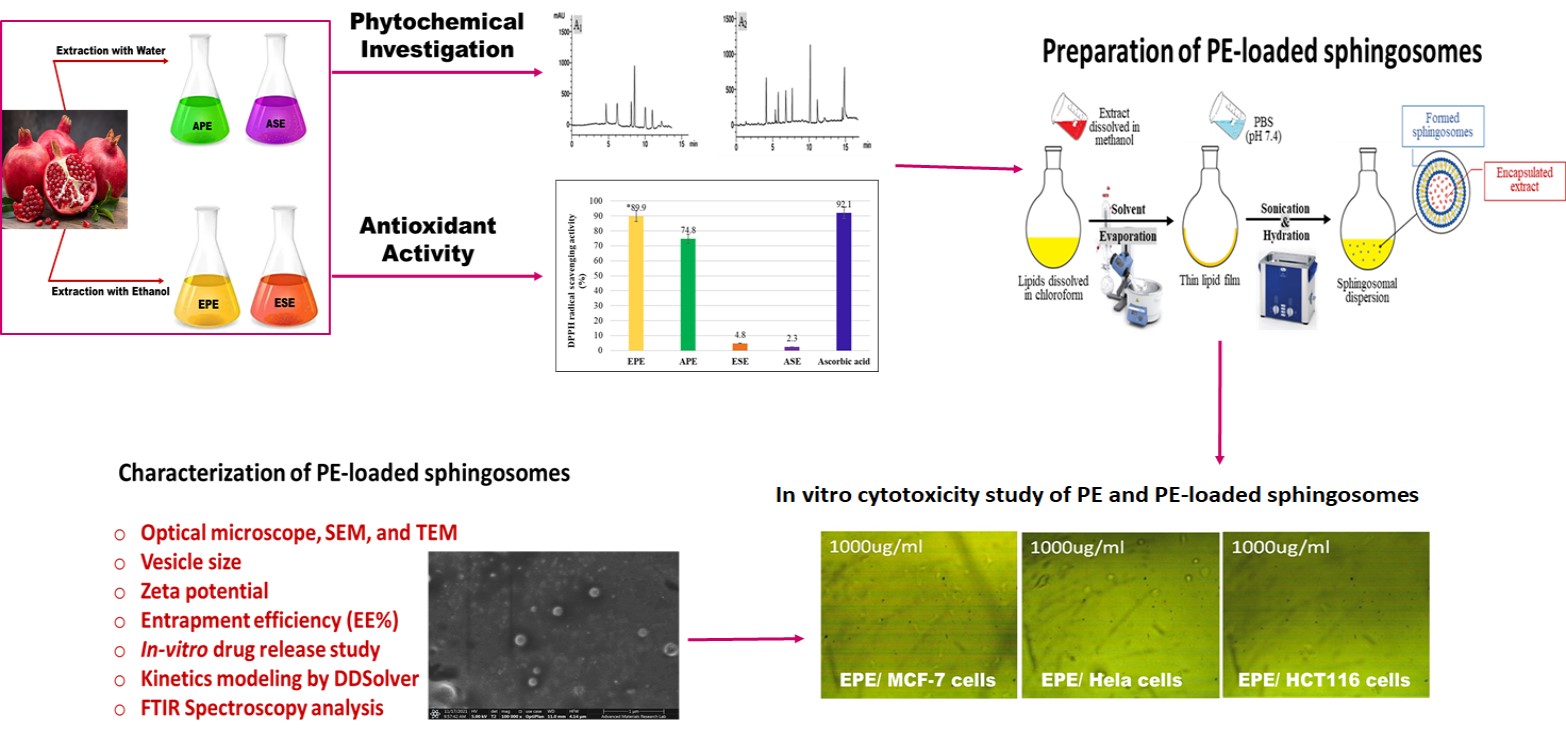

Supplement: S1 Graphical abstract — (JPG) [file pone.0293115.s001.jpg]
